# Synaptic gradients transform object location to action

**DOI:** 10.1038/s41586-022-05562-8

**Published:** 2023-01-04

**Authors:** Mark Dombrovski, Martin Y. Peek, Jin-Yong Park, Andrea Vaccari, Marissa Sumathipala, Carmen Morrow, Patrick Breads, Arthur Zhao, Yerbol Z. Kurmangaliyev, Piero Sanfilippo, Aadil Rehan, Jason Polsky, Shada Alghailani, Emily Tenshaw, Shigehiro Namiki, S. Lawrence Zipursky, Gwyneth M. Card

**Affiliations:** 1 Department of Biological Chemistry, Howard Hughes Medical Institute, David Geffen School of Medicine, University of California, Los Angeles, Los Angeles, CA USA; 2 Janelia Research Campus, Howard Hughes Medical Institute, Ashburn, VA USA; 3 Department of Computer Science, Middlebury College, Middlebury, VT USA; 4 Present Address: Research Center for Advanced Science and Technology, University of Tokyo, Tokyo, Japan; 5 Present Address: Department of Neuroscience, Howard Hughes Medical Institute, The Mortimer B. Zuckerman Mind Brain Behavior Institute, Columbia University, New York, NY USA

**Keywords:** Neural circuits, Sensorimotor processing

## Abstract

To survive, animals must convert sensory information into appropriate behaviours^1,2^. Vision is a common sense for locating ethologically relevant stimuli and guiding motor responses^3–5^. How circuitry converts object location in retinal coordinates to movement direction in body coordinates remains largely unknown. Here we show through behaviour, physiology, anatomy and connectomics in *Drosophila* that visuomotor transformation occurs by conversion of topographic maps formed by the dendrites of feature-detecting visual projection neurons (VPNs)^6,7^ into synaptic weight gradients of VPN outputs onto central brain neurons. We demonstrate how this gradient motif transforms the anteroposterior location of a visual looming stimulus into the fly’s directional escape. Specifically, we discover that two neurons postsynaptic to a looming-responsive VPN type promote opposite takeoff directions. Opposite synaptic weight gradients onto these neurons from looming VPNs in different visual field regions convert localized looming threats into correctly oriented escapes. For a second looming-responsive VPN type, we demonstrate graded responses along the dorsoventral axis. We show that this synaptic gradient motif generalizes across all 20 primary VPN cell types and most often arises without VPN axon topography. Synaptic gradients may thus be a general mechanism for conveying spatial features of sensory information into directed motor outputs.

## Abstract (web summary)

Analysis of behaviour, physiology, anatomy and connectomics in *Drosophila* shows how direction-specific visual information is transformed onto downstream premotor networks and converted into appropriate motor responses.

## Main

To catch a ball, turn when called or pick up a cup, our brains must direct not just what to do, but where to do it. Inherent to this process is a ‘sensorimotor transformation’^2,8,9^ in which an object’s location detected in sensory space, such as the position on the retina, is converted into movement direction in motor coordinates, such as the direction of limb or joint angle changes. There is considerable evidence that topographically organized brain regions in a wide range of species encode the location and identity of visual objects^10–13^; however, how neural connectivity patterns convey such information to downstream premotor networks, and how developmental programs specify this connectivity, remains poorly understood.

**Fig. 1:**
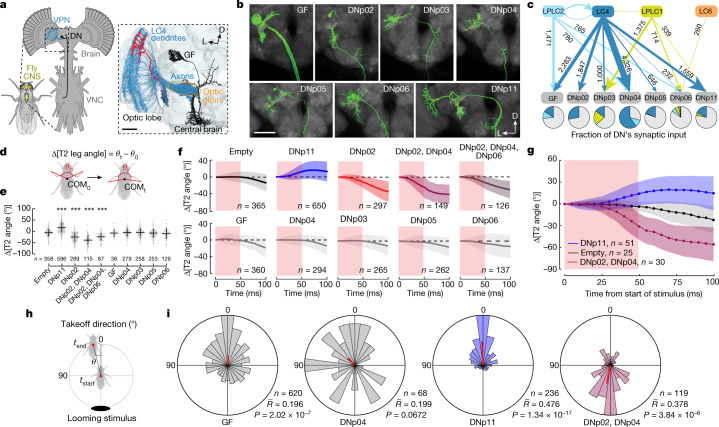
LC4 VPNs pass looming location information to DNs that mediate forward or backward escape takeoffs. **a**, VPNs with retinotopically arranged dendrites in the lobula neuropil of the fly optic lobe have axon terminals in cell-type-specific optic glomeruli in the central brain. Dendrites of >50 postsynaptic neurons typically innervate each optic glomerulus. Inset: EM-based reconstructions (hemibrain connectome^27^) of 71 LC4 VPNs (blue), a single LC4 neuron (red) and LC4 postsynaptic partner, GF DN (black). VNC, ventral nerve cord; D, dorsal; L, lateral; glom., glomerulus. Scale bar, 20 μm. **b**, Confocal projections of GFP (green) expression in seven DNs innervating the LC4 glomerulus (red dashed line). Grey, brain neuropils. Images adapted from ref. ^24^, CC BY 4.0 (*n* = 4 brains for each DN). Scale bar, 50 μm. **c**, Synaptic connectivity from looming-sensitive VPN cell types onto seven DNs based on the hemibrain connectome. Arrow width is proportional to synapse number. Pie charts indicate proportion of a given DN’s inputs from each looming-sensitive VPN cell type. **d**, Forward–backward postural shifts in response to DN photostimulation; quantified as Δ[T2 leg angle], the change in angle between the middle jumping legs and COM. **e**, Δ[T2 leg angle] 75 ms after the onset of 50-ms photostimulation. Points, individual flies; error bars, s.d.; one-way analysis of variance (ANOVA), Dunnett’s test, ***P < 0.001, exact *P* values in Supplementary Table 1. **f**, Δ[T2 leg angle] time courses from machine-learning-tracked data; red shaded area, photostimulation period. **g**, Δ[T2 leg angle] for a subset of manually annotated flies. In **f**,**g**: lines, mean; shading, s.d. **h**, Takeoff direction is COM movement direction between onset of middle leg extension and takeoff. **i**, Polar histograms of optogenetically activated takeoff direction. Red line, circular mean; *n*, number of flies tested; $\bar{R}$, mean vector length; *P*, Hodges–Ajne test for angular uniformity. Source data

In *Drosophila*, VPNs that have dendrites in the optic lobe and axon terminals in the central brain detect ethologically relevant visual features, such as small-object motion or looming of dark objects^6,7,14–17^, and are close to the sensorimotor interface. Multiple VPN types initiate visually guided behaviours^6,18–21^, and some VPN types synapse directly onto a subset of the ≈500 premotor descending neurons (DNs) per hemibrain whose activation drives distinct motor actions^22–24^. There are 20–30 different types of VPN, each a population of 20–200 neurons per hemibrain (Fig. 1a), with small receptive fields (20–40°) that together cover visual space^6,15,16^. VPN dendrites in the optic lobe thus form a topographic map of visual space, and object location on the fly’s retina is theoretically encoded by which VPN neurons within a given type are excited. However, it has been unclear whether, and how, this spatial information is passed to downstream partners because the axons of all VPNs within a given type terminate in narrow, distinct glomeruli within the central brain (Fig. 1a) with little^25^ or no^6,15,26,27^ observable topography at the light-microscopy level. Yet several VPN cell types have been associated with direction-specific behaviours, including backing up and turning, escaping looming stimuli from different directions, collision avoidance and, in flight, saccade turns away from a visual stimulus^6,28–30^. Here we examine how direction-specific visual information is transformed onto downstream premotor networks by exploring the VPN-to-postsynaptic partner interface using electron microscopy (EM), light microscopy, physiology and behaviour.

## Neural control of looming escape direction

**Extended Data Fig. 1:**
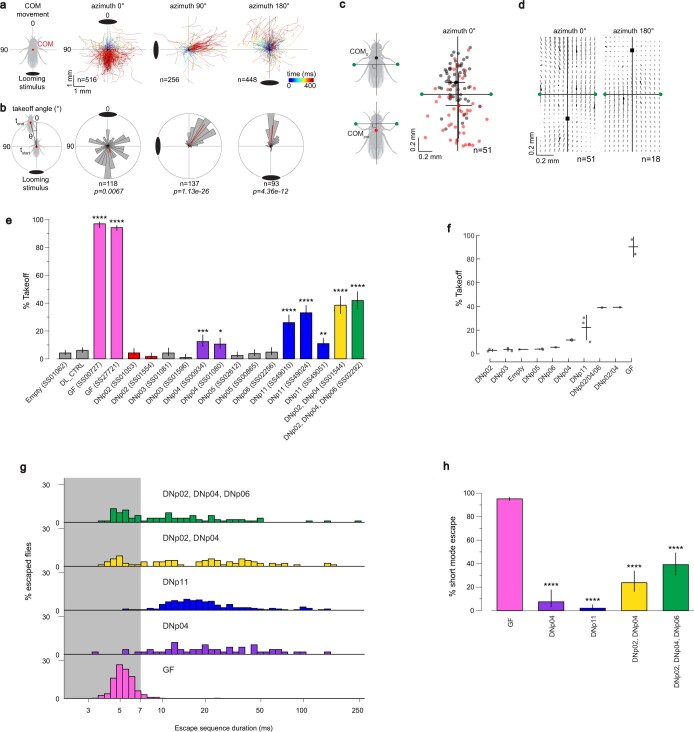
Control of fly takeoff direction. **a**, When shown a looming stimulus from the front (azimuth 0°), side (azimuth 90°), or back (azimuth 180°), flies respond by moving their center of mass (COM) away from the stimulus. Black disc represents stimulus location and color indicates time from stimulus onset. Flies used for **a**–**d** were a control genotype for other experiments, Empty>Kir2.1. One trial per fly. **b**, Some flies also takeoff in response to looming, and those that do takeoff in a direction away from the stimulus (with some influence of the heading of the fly^33^). Shown are polar takeoff direction histograms with 12° bin width and mean resultant vector overlaid (red line). p, Hodges-Ajne test for angular uniformity. **c**, Takeoff direction results from the fly shifting its COM relative to the axes formed by a line connecting the ground contact points of its two middle jumping legs and a perpendicular bisector. Black points indicate COM at stimulus onset and red points indicate COM just prior to takeoff. **d**, The specific direction in which the COM moves in body coordinates depends on its starting location. Vector position is the COM position at stimulus onset. The vector itself indicates the shift of COM position from stimulus onset to just prior to takeoff. Black vectors are tracked data, gray vectors are interpolated. Black square is approximated point of convergence. **e**, Percent of flies (individual DN driver lines) that performed a takeoff in response to CsChrimson optogenetic activation in the FlyPEZ assay. Error bars, Wilson score interval; *p < 0.05, **p < 0.01, ***p < 0.001, ****p < 0.0001 vs control (Empty, empty brain split-Gal4 control; DL – wild type control); normal approximation to binomial, two-sided Z-test, Bonferroni correction post hoc test. **f**, Same data as in (**e**) but with driver lines grouped by cell type. Error bars, SD. **g**, Histograms displaying the distribution of escape sequence durations between the wing raising and takeoff jump sub-behaviors (for LC4-DN driver lines expressing CsChrimson that can elicit escape upon activation). Escape trials are combined from split-Gal4 lines for each LC4-DN type. Short-mode escape duration (0 to 7 ms, gray shaded region) and long-mode escape duration (>7 ms), as previously established. **h**, Percentage of short-mode activated escapes. Error bars, Wilson score interval; ****p < 0.0001 versus GF; normal approximation to binomial, two-sided Z-test, Bonferroni correction post hoc test. Detailed description of statistical tests used and p-values for panels “e” and “h” is available in Supplementary Table 1. Source data

Looming visual cues indicate an impending collision or predator attack and drive rapid escape actions in most visual animals^31,32^. Flies orient their escape takeoff away from the direction of a looming stimulus^28,33^. Several *Drosophila* VPN types respond to looming stimuli^6,16,33,34^, in particular LC4, a population of about 60 neurons per hemibrain, whose activation is critical for fast escape takeoffs through direct synapses onto the giant fibre (GF) DN^35^ (Fig. 1a). To investigate the control of escape direction, we measured fly responses to three different directions of looming using the FlyPEZ^33^ automated assay and machine-learning-based automated tracking (Extended Data Fig. 1a). Flies moved their centre of mass (COM) away from the stimulus direction (Extended Data Fig. 1a), and takeoffs were generally^33^ away from the stimulus (Extended Data Fig. 1b). As previously suggested^28^, we found takeoff direction arose from pre-takeoff postural shifts of a fly’s COM relative to its middle pair of legs (Δ[T2 leg angle]; Extended Data Fig. 1c,d), which power the takeoff jump. This indicates that object location encoded by looming-sensitive VPNs, such as LC4, is passed downstream.

GF activation does not drive postural adjustments^36^ and is not expected to control the escape takeoff direction. LC4 axons, however, overlap with dendrites of nine other DNs^24^ (here called LC4-DNs). To examine whether LC4-DNs control takeoff direction, we focused on seven for which we had DN-specific genetic driver lines^24^ (Fig. 1b). Analysis of the *Drosophila* ‘hemibrain connectome’, reconstructed from EM data^27^, confirmed that these DNs receive direct visual input from looming-sensitive VPNs, and (except for DNp06) a substantial portion of this is from LC4 (Fig. 1c) with four of them (DNp04, GF, DNp02 and DNp11) among the top 10 downstream partners of LC4 (ref. ^27^). We optogenetically activated each DN, as well as two ‘combination’ lines targeting either two or three LC4-DNs together, and analysed the resulting behaviour with high-speed video^33^. GF activation produced takeoff rates of greater than 90% (refs. ^33–36^). Only DNp04, DNp11 and combination line activation increased takeoff rates significantly compared to that of controls (Extended Data Fig. 1e,f and Supplementary Table 1), albeit with rates lower than that for GF activation (that is, 15–40% versus >90%), suggesting that natural threats may simultaneously activate multiple LC4-DNs to drive downstream escape motor circuits. DNp04- and DNp11-activated takeoffs were almost exclusively ‘long-mode’, in which the wings are raised before the takeoff jump, whereas GF activation produced ‘short-mode’ escapes without prior wing-raising as previously described^36^ (Extended Data Fig. 1g,h and Supplementary Table 1). Combination line activation drove primarily long-mode takeoff, but did also unexpectedly produce many short-mode takeoffs, which are thought to rely on GF activation. Taken together with the findings of our previous work^37^, this mixed result indicates either that the combination of DNp02, DNp04 and DNp06 inputs to the GFs, or that these DNs are not naturally co-activated with the strong intensity of optogenetic activation.

To evaluate whether any of these DNs triggered postural adjustments critical for escape directionality, we tracked 11 body points using Animal Part Tracker software (Branson Lab, see Methods) and created a metric for postural shift (Fig. 1d). DNp11 activation drove flies to lean forwards, whereas activation of DNp02 (including combinations of DNp02 and DNp04 or DNp02, DNp04 and DNp06) promoted backward leaning (Fig. 1e–g and Supplementary Videos 1 and 2). We next assessed whether these induced postural shifts led to directional takeoffs (Fig. 1h,i). Activation of DNp11 evoked forward takeoffs (Fig. 1i), whereas activation of DNp02 and DNp04 together evoked a strong bias towards backward takeoffs (Fig. 1i). As activation of DNp04 alone resulted in omnidirectional takeoffs (Fig. 1i), we reasoned that DNp02 was the main contributor to the movements leading to backward takeoff. The weak forward takeoff bias from GF activation probably results from the average resting posture of the fly, which was previously observed to have the COM slightly in front of the T2 legs^28^.

**Extended Data Fig. 2:**
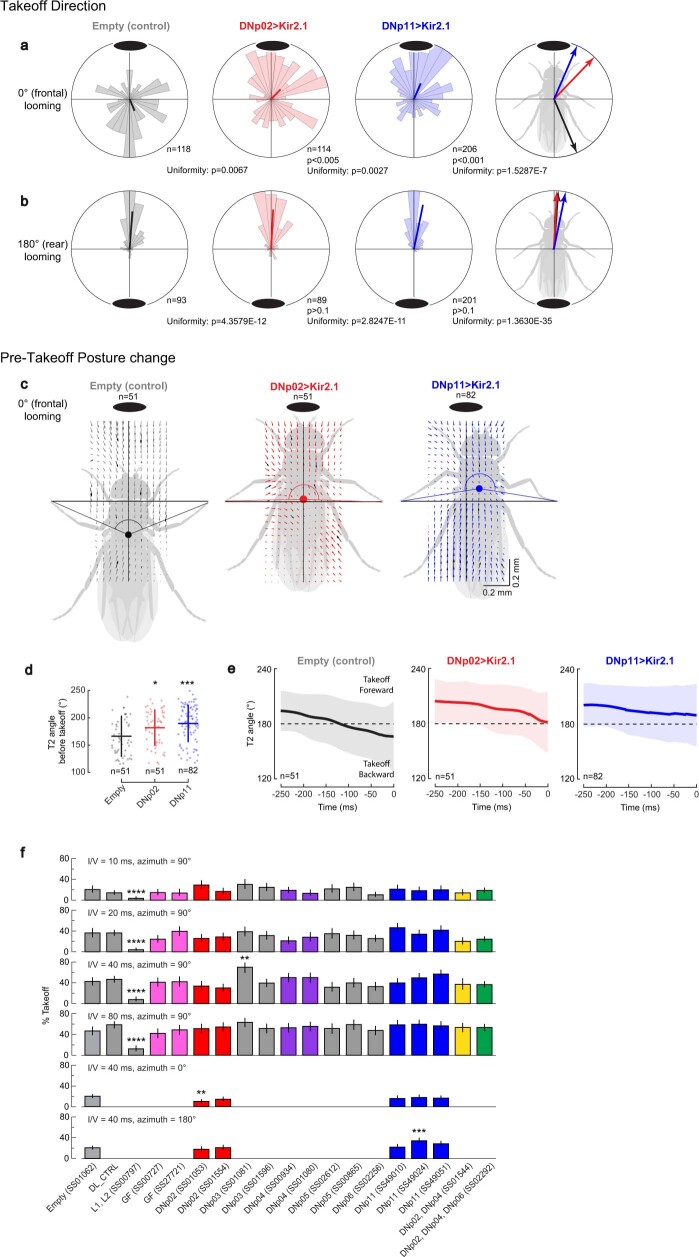
Silencing of either DNp02 or DNp11 impairs control of postural shifting and takeoff direction in response to looming stimuli. For neuronal silencing experiments, driver lines for DNp02, DNp11, and an ‘Empty’ driver line control were crossed into UAS-Kir2.1. **a**–**b**, Polar takeoff direction histograms in response to looming stimuli presented in front of the fly at 0° azimuth (**a**) or behind the fly at 180° azimuth (**b**); 12° bin width; red line, mean resultant vector. DN-silenced flies perform normally in response to a posterior (180°) stimulus compared to control (p > 0.1 for both DNs, Kuiper’s Test). However, DN silencing altered the distribution of backward takeoffs direction in response to frontal looming (0°) for both DNp02 (p < 0.005, Kuiper’s test) and DNp11 (p < 0.001, Kuiper’s test) silencing compared to controls. Strikingly, many DNp02- and DNp11-silenced flies performed forward takeoffs in response to frontal looming stimulation, effectively jumping toward the threatening stimulus. **c**, To further understand why flies were inappropriately taking off forwards, we looked at how much DN-silenced flies moved their COM backwards in response to 0° looming. We visualized COM movement in body coordinates from different starting postures using the same flow fields in body-centric coordinates as in Extended Data Fig. 1d. Visual inspection indicated that COM movement fields for DN-silenced flies differed from controls in the amount of backwards movement and had more lateral movement. **d**, To quantify this motion, we measure the T2 angle (angle formed by T2 tarsal contact points and COM), which is >180° when the COM is in front of the T2 jumping legs and <180° when the COM is behind the T2 jumping legs. The mean T2 angle just before takeoff was significantly different for DNp02- and DNp11-silenced flies compared to controls (*p = 0.0468, ***p = 4.79e-04, One-Way ANOVA, Dunnett’s test). Black points, individual flies; error bars, SD. **e**, Looking at time courses for T2 leg angle in response to 0° azimuth looming stimulus for the different DN-silenced lines (colors, shaded area, SD), with control data overlaid (grey), it is clear that the difference in the DN-silenced flies is that they do not shift backwards as much as controls. Since COM placement prior to takeoff determines whether the fly’s jump will propel it forwards (T2 angle>180) or backwards (T2 angle<180), the impaired pre-takeoff T2 leg angle change in DNp02- and DNp11-silenced flies, which on average does not become <180° as in control flies, likely underlies altered takeoff performance leading to more forward-directed takeoffs. **f**, DNp02 and DNp11 silencing does not affect takeoff rates. Percentage of flies which performed a takeoff to a looming visual stimulus (azimuth = 90°, elevation = 45°) at four looming rates (l/v = 10, 20, 40 and 80 ms), or a looming visual stimulus (azimuth = 0° or 180°, elevation = 45°) at l/v = 40. L1/L2-silenced flies serve as “motion-blind” negative controls. Error bars, SEM; Wilson score interval; **p < 0.01, ***p < 0.001, ****p < 0.0001 versus Empty control; normal approximation to binomial, two-sided Z-test, Bonferroni correction post hoc test. Detailed description of statistical tests and p-values for panel “f” is available in Supplementary Table 1. Source data

To further test whether DNp02 and DNp11 contribute to directional control during looming-evoked escape, we silenced each DN by selectively expressing Kir2.1, an inwardly rectifying potassium channel, and then measured responses to frontal (0°) or rear (180°) looming stimuli (Extended Data Fig. 2). DNp02-silenced flies took off normally (forwards) in response to rear stimuli but showed significant impairment in their ability to take off backwards in response to frontal stimuli—on average most DNp02-silenced flies took off forwards, directly towards the stimulus. This is consistent with the activation of DNp02 driving a backward postural shift, and supports a critical role for DNp02 in the postural adjustments that control backward takeoffs. Notably, flies in which DNp11 was silenced had a similar phenotype—these flies took off forwards in response to both frontal and rear looming stimuli. This could indicate that more DNs, possibly with interconnections, are involved in the control of forward takeoffs than backward ones, and also probably reflects the bias of the fly to jump forwards if no postural adjustment is made from the common resting posture. We conclude that, as flies with either DNp11 or DNp02 inactivated did not respond with normal takeoff directions to anterior or posterior looming stimuli, both DNs contribute to directional control of the fly’s natural escape behaviour.

## EM reveals LC4-to-DN synaptic gradients

**Fig. 2:**
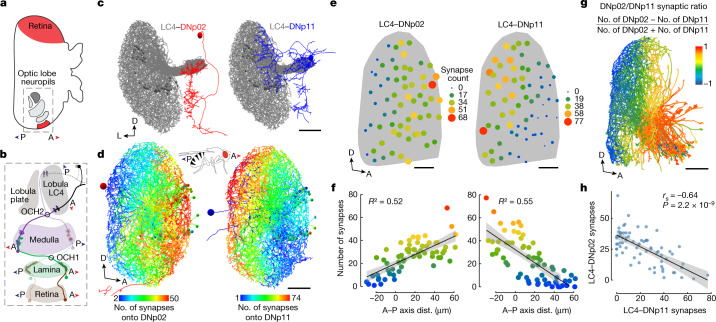
Synaptic number gradients between LC4 and DNs transform a retinotopic map in the optic lobe to movement direction. **a**, Fly visual system (dorsal view). The A–P axis of the visual space is mapped onto the anatomical lateral–medial axis of the lobula neuropil. The outlined area is shown in **b**. **b**, Anterior and posterior visual inputs to LC4 neurons through two optic chiasms (OCHs). Images in **a**,**b** adapted from ref. ^50^, CC BY 4.0. **c**, DNp02 (red) and DNp11 (blue) dendrites receive input from LC4 neurons (grey) in the glomerulus formed by LC4 axon terminals. Shown are neuron skeletons (red and blue). Scale bar, 50 μm. **d**, LC4 dendrites in the lobula (lateral view) colour-coded according to the number of synapses their axons make onto DNp02 or DNp11. LC4–DNp02 and LC4–DNp11 synaptic gradients are antiparallel along the A–P axis of the visual space. Scale bar, 20 μm. All neurons in **c**,**d** are manually reconstructed from the EM FAFB dataset. **e**, Antiparallel A–P gradients are also seen in the hemibrain connectome. Dots, two-dimensional (2D) lobula projections of dendritic centroids for individual LC4 neurons in the lobula weighted in size and colour by the number of synapses made by their axons onto DNp02 and DNp11. Scale bars, 25 μm. **f**, Regression of LC4-DN synaptic weights as a function of LC4 dendrite centroid location; colour as in **e**. Linear fit line overlaid. Error bands, s.e.m. **g**, Hemibrain connectome reconstruction of LC4 dendrites coloured on the basis of a normalized (−1 to 1) number of synapses each LC4 neuron forms with DNp02 and DNp11. Some anterior lobula dendrites exceed the EM volume and are not fully reconstructed. **h**, Correlation between the number of synapses each LC4 neuron (*n* = 71) makes with DNp02 and DNp11. *r*_s_, Spearman’s rank correlation coefficient. A, anterior; P, posterior; D, dorsal; L, lateral. Error band, s.e.m. Source data

**Extended Data Fig. 3:**
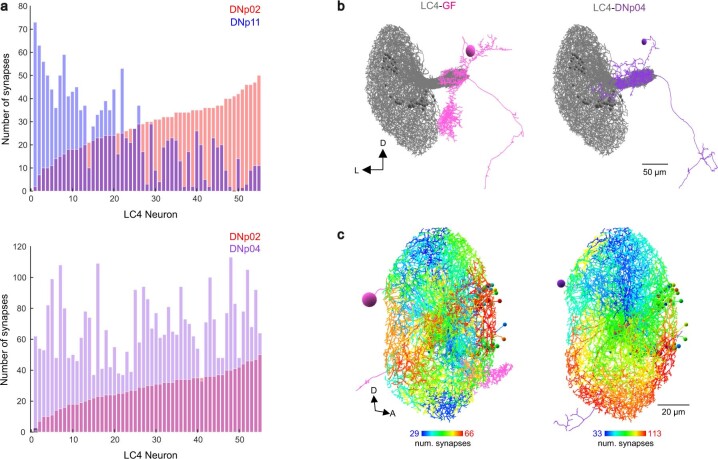
EM-based analysis if synaptic connectivity patterns between LC4 and DNs. **a**, Individual LC4 neurons (55 total traced in “FAFB” EM volume) make different numbers of synapses with different DNs. Individual LC4 neurons (x-axis) are ordered according to the synapse number with DNp02 (red bars). LC4 synapse number with DNp11 (blue bars) and DNp04 (purple bars). **b**, LC4 neurons (grey) and synaptic partners (GF and DNp04) traced in a FAFB EM volume. DN soma, circles. **c**, LC4 dendrites (lateral views) color-coded according to the total number of synapses onto each DN. D, dorsal; L, lateral; A, anterior. Source data

We next sought to determine how LC4 neurons differentially convey the spatial location of the looming stimulus to DNp11 and DNp02 (Fig. 2a,b). In the right hemisphere of a complete serial section transmission EM dataset, we traced all LC4 neurons, DNp02 and DNp11 (FAFB dataset^38^; Fig. 2c) and marked synapses between LC4 neurons and each DN. We found a wide range (1 to 75) in the number of synapses individual LC4 neurons made with a given DN (Extended Data Fig. 3a). We next investigated whether LC4 neurons that synapsed more with DNp11 or DNp02 had dendrites located in a particular region of the lobula neuropil. We visualized the LC4 dendrites in the lobula and coloured each neuron by the number of synapses it made with a given DN. This revealed antiparallel synaptic number gradients along the lobula anterior–posterior (A–P) axis for DNp02 and DNp11 (Fig. 2d). By contrast, A–P gradients were not seen in LC4 connectivity onto the GF and DNp04 (Extended Data Fig. 3b,c). The same A–P gradient patterns with LC4 synapses onto DNp11 and DNp02 were seen in an EM dataset from a second brain (hemibrain)^27^ (Fig. 2e–g). This was supported by a strong negative correlation between the number of synapses a given LC4 makes with DNp11 and with DNp02 (Fig. 2h). The orientation of these gradients corresponds to the backward- and forward-jumping motor outputs of DNp02 and DNp11, respectively.

Taken together, the behaviour and connectomic data support a simple model: antiparallel synaptic gradients transform locally detected object location into oppositely directed behaviours. A frontward looming stimulus activates anterior LC4 neurons that provide relatively more drive to DNp02, which produces backward body movements generating a backward escape trajectory following co-activation with DNp04 or other escape pathways. For a stimulus looming from behind, posterior LC4 neurons become more active and drive DNp11 to generate forward postural shifts and a forward-directed takeoff.

## Synaptic gradients are functional

**Fig. 3:**
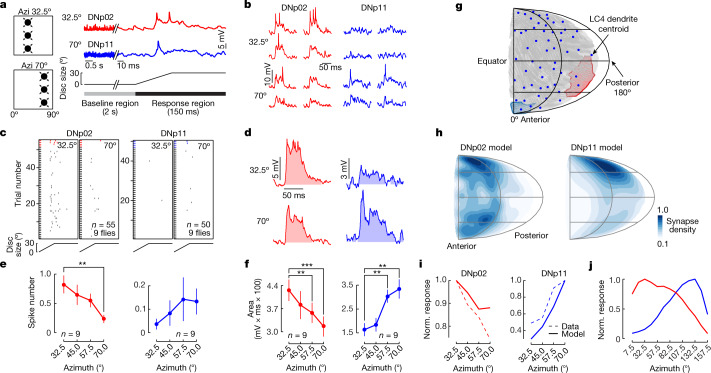
LC4 synaptic number gradients onto DNp02 and DNp11 are functional. **a**, Whole-cell electrophysiological recordings of DNp02 (red) and DNp11 (blue) to looming stimuli at 32.5° (for DNp02) and 70° (for DNp11) in azimuth. Looming stimulus is an array of three discs expanding 0° to 30° diameter at 500° s^−1^. Shown are representative traces from a single fly and stimulus. **b**, Representative responses from a single fly for 32.5° (top) and 70° (bottom) azimuth looming stimuli. **c**, Spike raster plots of DN responses during the 150 ms after looming onset. Coloured trials show the traces in **b**. **d**, Averaged response of the traces in **b** shows subthreshold depolarizing responses to looming stimuli. Shaded area, estimated depolarization from the baseline. **e**, Mean per-trial spike count across individual flies (from **c**). *n*, individual trials; **P < 0.01. **f**, Pooled mean of integrated potentials across individual flies. *n*, individual trials. Repeated-measures one-way ANOVA, Dunnett’s test. Error bars, s.e.m.; **P < 0.01, ***P < 0.001, see Supplementary Table 1 for exact *P* values. **g**, Mollweide projection of estimated dendritic receptive fields for all 55 LC4 neurons in the FAFB EM dataset. Polygons are estimated visual fields of individual LC4 neurons (example individual fields in red and blue). **h**, DNp02 and DNp11 LC4-receptive fields estimated on the basis of summed input from individual LC4 fields in **g**. **i**, In vivo whole-cell (dashed) and model-estimated (solid) DN responses to three-loom-array stimuli (solid). **j**, Estimated DNp02 and DNp11 responses to modelled three-loom-array stimuli across the whole visual hemifield, based on receptive fields in **h**. Source data

**Extended Data Fig. 4:**
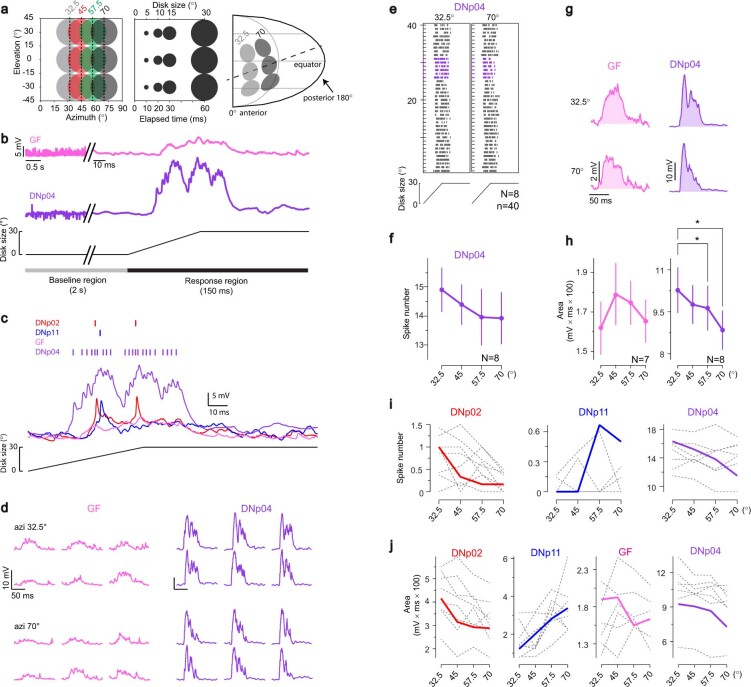
In vivo whole-cell recordings from DNs upon looming stimulation. **a**, Left: Schematic of looming array stimuli at different azimuths (32.5°, 45°, 57.5° and 70°). Each loom consists of three dark disks in the white background. Pseudo-colored for clarity. Middle: looming array disk size over time from the beginning of stimulus. Each disk expands from 0° to 30° at 500°/s. Right: Stimulus arrangement projected onto fly’s eye (0° front of fly). To align looming array stimuli more closely with the synaptic gradients, the whole plane of the stimuli was pitched down 20°. **b**, Top: whole-cell electrophysiological recordings of the GF and DNp04 to looming stimuli at 32.5° in azimuth. Shown are representative traces from a single fly and stimulus. Middle: change of disk size over time. Bottom: baseline region and response region defined in the traces for analysis of DN activity. **c**, Representative DN responses showing identified spikes (top rasters). **d**, Representative responses from a single fly for 32.5° (top) and 70° (bottom) azimuth looming stimuli. Representative traces for GF and DNp04 are from a single fly with 6 trials to each stimulus. **e**, Spike raster plots of DNp04 in 150 ms time window after the onset of looming stimuli. **f**, Pooled mean of DNp04 spike numbers across individual flies (from “e”). Error bars, SEM; RM one-way ANOVA, Dunnett’s test. **g**, Averaged response of the representative traces in **d** shows subthreshold depolarizing responses to looming stimuli. Shaded area under the line shows estimated depolarization from the baseline. **h**, Pooled mean of integrated potentials for DNp04 and GF across individual flies. Error bars, SD; RM one-way ANOVA, Dunnett’s test. **i**–**j**, Mean spike numbers (**i**) and mean of integrated potentials (**j**) across trials in individual flies in response to looming stimuli. Colored lines denote the representative traces of each DN in Fig. 3a and Extended Data Fig. 4b. Detailed description of statistical tests and p-values for panels “f, h” is available in Supplementary Table 1. Source data

The synapse gradient model is based on the assumption that synapse number correlates with connection strength. To directly test this, we carried out in vivo whole-cell patch-clamp recordings from DNp02, DNp11, DNp04 and the GF during visual looming stimulation at varying locations along the A–P axis of the visual space. We presented vertical arrays of small dark expanding discs at four different azimuthal locations ipsilateral to the targeted DN (Fig. 3a and Extended Data Fig. 4a). DNp02, DNp11, the GF and DNp04 all depolarized in response to looming, and all except the GF produced action potentials (Fig. 3a–f and Extended Data Fig. 4b–j; see Methods for identification of action potentials).

DNp02 produced more action potentials in response to anterior, compared to more posterior, stimuli (44 versus 13 spikes across all trials), whereas DNp11 exhibited the opposite trend (Fig. 3b,c,e and Extended Data Fig. 4i). These trends were consistent for both individual (Fig. 3c and Extended Data Fig. 4c,i) and averaged (Fig. 3e) responses. By contrast, DNp04 produced bursts of action potentials without significant azimuth tuning (Extended Data Fig. 4c–f,i). In agreement with the action potential tuning curves, depolarizing membrane potentials in DNp02 were larger for more anterior azimuthal locations of the looming stimulus, whereas those for DNp11 were larger for more posterior looming locations (Fig. 3d,f and Extended Data Fig. 4j). For the GF, we did not see distinct tuning properties for the anterior–posterior location of the stimuli. DNp04 did show a trend towards stronger responses to anterior stimuli, although the responses were more variable than for DNp11 or DNp02 (Extended Data Fig. 4g,h,j).

**Extended Data Fig. 5:**
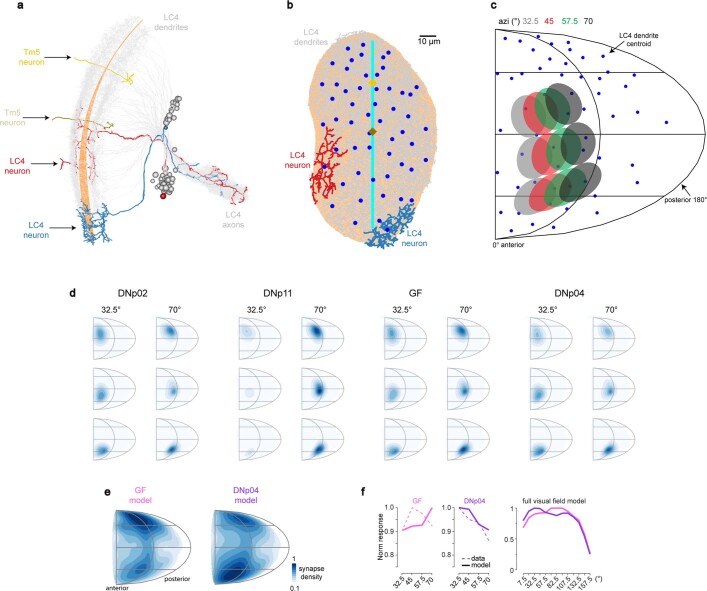
EM reconstruction of LC4 neurons and estimated receptive fields. **a**, Tracing of all 55 LC4 neurons on one side of the brain in FAFB EM volume. Two example neurons are colored in red and blue (same in “b”). LC4 neurons have dendrites in lobula layers 2 and 4. LC4 cell bodies are marked with grey circles. Two Tm5 neurons are chosen to determine the center and central meridian of the eye in “b”. **b**, Two-dimensional projection of the lobula layer. Traced LC4 dendrites (grey) are projected onto a surface fit through all dendrites (orange). Blue circles denote centers of mass of individual LC4 dendrites. Vertical line is the estimated central meridian that divides the eye between anterior and posterior halves. **c**, Pseudo-stimuli mimicking looming stimuli used in in vivo whole-cell recordings to simulate LC4DN responses based on LC4 connectivity. **d**, Confined DN RFs to the region of pseudo-stimuli in the eye coordinate imitating the looming stimuli. LC4 RFs based on connectivity to each DN are scaled by the proportion of overlapping with each stimulus region prior to sum up all the LC4 RFs. **e**, Estimated anatomical receptive fields (RFs) of the GF and DNp04 in the eye coordinates. **f**, Left: Estimated connectivity-based DN responses to pseudo-stimuli (solid) and measured responses to the corresponding looming stimuli (dashed) from *in vivo* whole-cell recordings. Right: Estimated responses in DNp04 and GF extrapolated using the connectivity model to looming pseudo-stimuli that cover the visual hemifield.

If synapse number correlates directly with input current drive to the postsynaptic cell, we should be able to predict the DN responses to looming stimuli at different azimuthal locations. To assess this, we used the EM data to make a model incorporating both the spatial profile of LC4 dendrites and the synaptic connectivity of LC4 axons with DNs. Main dendritic branches of all 55 LC4 neurons in the FAFB dataset^38^ were mapped from lobula to eye coordinates following a previously established method^25^ (Fig. 3g and Extended Data Fig. 5a–c). The normalized estimated responses to looming recapitulated the azimuthal tunings predicted by the synaptic gradients and matched the responses for all four DNs we measured (Fig. 3h,i and Extended Data Fig. 5d–f). We conclude that the synaptic numbers observed from EM data can be interpreted as functional synaptic weights.

We used this model to simulate responses to looming from azimuthal locations across the whole visual hemisphere, including those not possible in our physiology experiments. Our simulation showed strong antiparallel looming response profiles for DNp02 and DNp11 across nearly the whole visual hemifield (30°–130°), supporting the observed synaptic gradients as predictive of functional response profiles (Fig. 3j). Taken together, these results corroborate the model that anterior LC4 neurons provide stronger inputs to DNp02 in response to anterior stimuli whereas posterior LC4 neurons provide more drive to DNp11 in response to posterior stimuli in a graded fashion. This differential connectivity drives the backward (DNp02) or forward (DNp11) escape takeoffs away from looming threats.

## Synaptic gradients are a common wiring motif

**Fig. 4:**
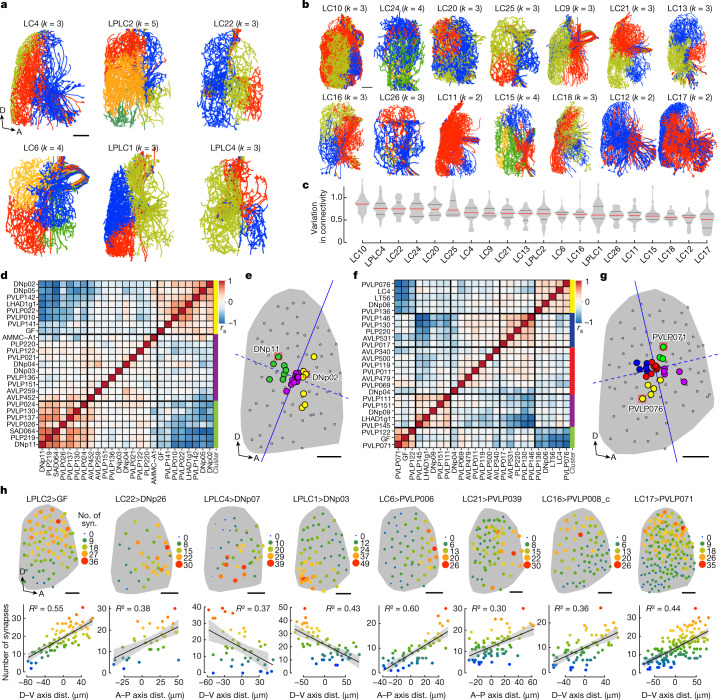
Synaptic gradients are a general property of VPN output organization. **a**,**b**, Connectivity-based *k*-means clustering of individual neurons within 20 VPN cell types (see Methods). Lateral views of VPN dendrites in the lobula (hemibrain connectome reconstructions). Individual cells within one VPN cell type are coloured by their cluster identity. Colours do not correspond between VPN types. Most VPNs exhibit distinct spatial separation (examples in **a**), but in some cases (LC12 and LC17 in **b**) there is no clear separation. Scale bars, 20 μm. **c**, Differential connectivity (number of synapses) across individual neurons within one VPN cell type. Measured for 20 VPN cell types and their postsynaptic partners that make at least 50 synapses total. Coefficients of variation in synapse number are averaged across all postsynaptic partners per VPN cell type. **d**, Matrix of pairwise correlations in synaptic connectivity between LC4 and its top 25 postsynaptic partners; ordered by hierarchical clustering as indicated by coloured side bars; *r*_s_, Spearman’s rank correlation coefficient. **e**, Topographic map of input centroids, weighted by number of synapses, for top 25 postsynaptic partners of LC4. Dark grey shading, lobula 2D projection; small open circles, centroids of 71 individual LC4 dendrites; coloured circles, weighted input centroids; solid blue line, median separation line; dashed blue line, projection line (see Methods). Red squares indicate centroids of DNp02 and DNp11. Scale bar, 25 μm. **f**,**g**, Similar analysis as in **d**,**e**, but for LPLC2. Red squares, centroids of neurons PVLP071 and PVLP076. Scale bar, 25 μm. **h**, Representative examples of synaptic gradients reflecting A–P and D–V axes of dendritic maps in multiple VPN cell types. syn., synapses. Scale bars, 25 μm (images 1 and 6–8) and 30 μm (images 2–5). A, anterior; P, posterior; D, dorsal; V, ventral. Error bands, s.e.m. See legend for Fig. 2c,d. Source data

**Extended Data Fig. 6:**
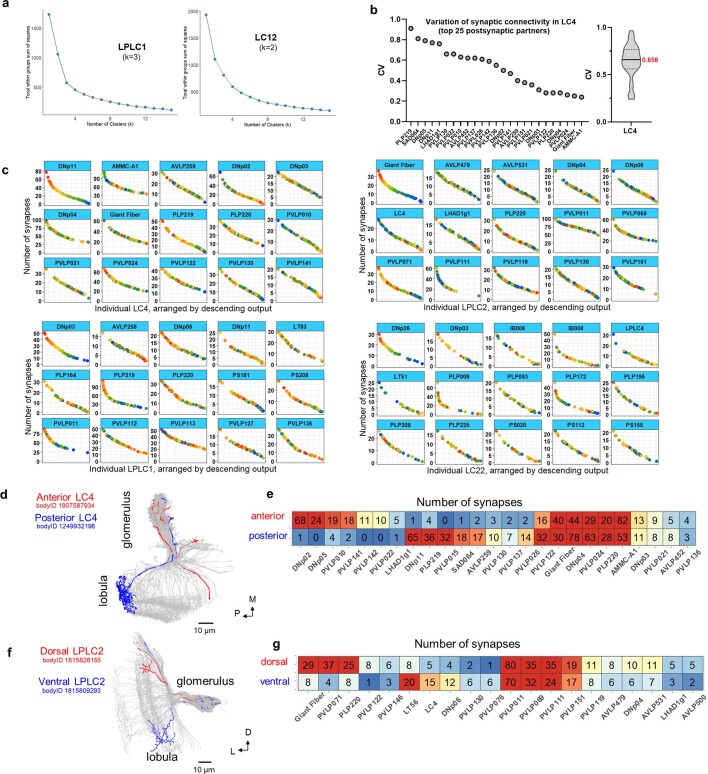
Differential synaptic connectivity in VPNs manifests as synaptic gradients reflecting visual space map. **a**, Representative elbow plots from k-means clustering of individual cells within each VPN cell type (based on the number of synapses they make with postsynaptic partners). A drop in the within cluster sum of squared distance was used to determine the number of k in Fig. 4a, b (see Methods for details). **b**, Representative example of differential synaptic connectivity metric (median coefficient of variation) for LC4. Left: coefficient of variation in synapse number between individual neurons within LC4 population and top 25 postsynaptic partners of LC4 (ordered by decreasing variation). Right: summary (median CV, shown in red) metric for all postsynaptic partners of LC4 making >50 synapses total. **c**, Representative examples of graded synaptic connectivity between four VPN cell types and their top 15 postsynaptic partners based on the total number of synapses. Each individual neuron within a VPN cell type is assigned a color based on just one plot (DNp11 for LC4, Giant Fiber for LPLC2 etc.), with the colors preserved in other graphs. Every plot indicates the number of synapses between individual neurons within one VPN cell type and a given postsynaptic partner (arranged by descending number of synapses). **d**, Single LC4 neurons (EM-based connectome reconstructions) with dendrites in anterior (bodyID 1907587934) or posterior (bodyID 1249932198) regions of the lobula are highlighted. The remaining LC4 neurons shown in grey. **e**, Differential synaptic connectivity between two LC4 neurons from (**d**) and their top 25 postsynaptic partners (measured by total number of synapses). 15 out of 25 postsynaptic neurons receive preferential or exclusive input from either anterior or posterior LC4. **f**–**g**, Differential synaptic connectivity of individual LPLC2 neurons with dendrites in dorsal (bodyID 1815826155) vs ventral (bodyID 1815809293) lobula. Similar to (**d**, **e**). P, posterior; M, medial; D, dorsal; L, lateral.

To address the question of whether visuomotor transformation through gradients of synapses is limited to just LC4 and DNp02 and DNp11 or whether it represents a general circuit wiring logic, we analysed the output connectivity patterns of 20 VPN cell types^6^ using data from the hemibrain connectome^27^. First, we used principal component analysis and *k*-means analyses to cluster individual neurons within a VPN cell type on the basis of the similarity of their outputs (that is, the number of synapses they form onto the set of synaptic partners within their respective optic glomerulus; Extended Data Fig. 6a and Methods). Next, we colour-coded each cluster to visualize the relationship between a neuron’s cluster identity and the spatial location of its dendrites in the lobula. A striking spatial separation of the clusters was found in most VPN cell types (Fig. 4a,b), revealing widespread differential synaptic connectivity, such that individual neurons within one VPN cell type elaborated quantitatively and qualitatively different outputs in the glomerulus depending on the location of their dendrites in the lobula (Fig. 4c and Extended Data Fig. 6b–g).

To investigate these properties in more detail, we analysed synaptic connectivity between two VPN cell types (LC4 and LPLC2) and the top 25 postsynaptic partners of each of them (Fig. 4d–g). Both VPN cells types are looming detectors and share some postsynaptic partners, including the GF^6,34,39^. For each VPN cell type, we first assessed the similarity of its outputs onto different postsynaptic neurons by measuring the pairwise correlation for all 300 possible pairs of its top 25 postsynaptic partners (similar to LC4 and DNp02 or DNp11 in Fig. 2h). The resulting matrices revealed that postsynaptic targets of LC4 and LPLC2 formed three and five connectivity-based clusters, respectively (Fig. 4d,f). Thus, different postsynaptic partners receive different patterns of input from the same VPN cell type. Next, to visualize the relationship between this differential input and VPN dendritic maps, we calculated weighted dendritic input centroids for each of the top 25 postsynaptic partners of LC4 and LPLC2, and measured pairwise distances between them (Extended Data Fig. 9a–h and Methods). These indicate spatial regions of the lobula providing the most input to a given postsynaptic partner. The resulting topographic maps (Fig. 4e,g) revealed that all three connectivity-based clusters for LC4 clearly segregated along the A–P axis of the lobula (Fig. 4e). By contrast, two out of five clusters for LPLC2 segregated along the A–P axis of the lobula, two segregated along the D–V axis, and one cluster had no spatial bias (that is, neurons from this cluster receive uniform input from all LPLC2 neurons; Fig. 4g). Notably, both the numbers and topographic positions of these clusters largely match the results of *k*-means analysis for both VPN cell types (Fig. 4a).

**Extended Data Fig. 7:**
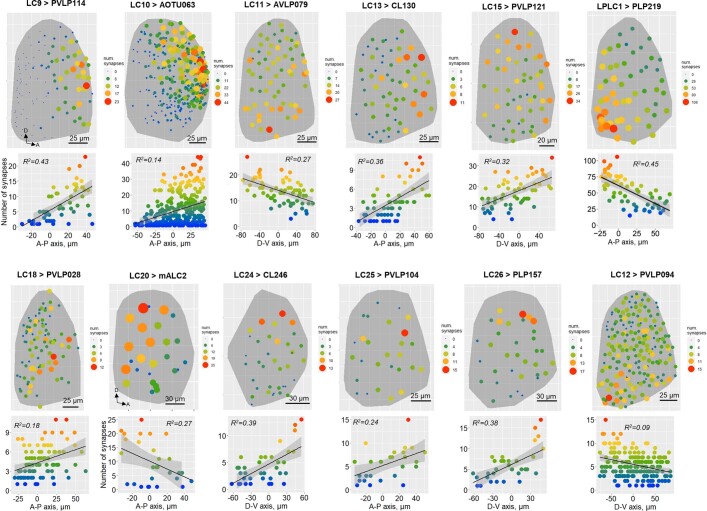
Synaptic gradients are a common wiring motif across 20 VPN cell types. Additional examples of synaptic gradients reflecting the visual space map between different VPN cell types and their postsynaptic partners. See legend in Fig. 2c,d. Top: 2D lobula projections of dendritic centroids for individual VPNs in the lobula weighted in size and color by the number of synapses made by their axons onto a designated postsynaptic target. Bottom: regression of synaptic weights as a function of VPN dendrite centroid location along the AP or DV axis in the lobula. Linear fit line overlaid. Error bands, SEM. low R^2^ value of LC10 gradients may be explained by the fact that LC10 bodyID annotations in the hemibrain EM volume contain multiple LC10 subtypes (LC10a-d) with differential synaptic specificity. D, dorsal; A, anterior.

These examples illustrate how the topographic map of VPN dendritic inputs in the optic lobe is converted into maps of graded synaptic weights in the optic glomerulus. We observed synaptic gradients reflecting both the A–P and D–V axes of the dendritic map across all 20 VPN cell types (Fig. 4h and Extended Data Fig. 7), analogous to those we originally found in the fly directional escape circuit (Fig. 2). The ethological relevance of some of these gradients may be deduced from the known function of postsynaptic neurons in the literature. For example, the D–V gradient from LPLC4 onto DNp07 may control landing behaviour^22^ (Fig. 4h) and the A–P gradient from LPLC1 onto PLP219 (Extended Data Fig. 7) could regulate collision avoidance^29^. Thus, we propose that conversion of visual space coordinates into graded synaptic connectivity is a shared feature of VPN wiring.

## Synaptic gradients with or without axon topography

**Fig. 5:**
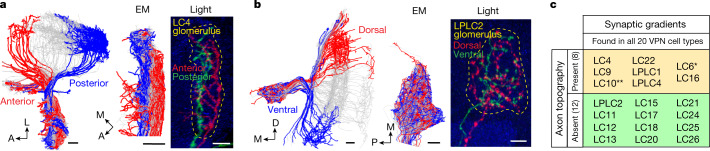
Axon topography is present in some, but not most, optic glomeruli. **a**, LC4 is a VPN cell type that retains axonal topography in optic glomerulus. Left, hemibrain connectome reconstructions of 15 anterior (red), 15 posterior (blue) and central LC4 cells (grey). Middle, EM reconstruction of axons in the LC4 glomerulus shows separation of anterior and posterior terminals. M, medial. Right, image of the LC4 glomerulus region with axon terminals of one anterior (red) and one posterior (green) cell labelled using MultiColor FlpOut and assessed using light microscopy (*n* = 9, all A–P pairs of individual clones from different brains exhibited reproducible axon terminal topography). Axonal projections form a topographic map in the glomerulus, corresponding to the location of their dendrites along the A–P axis of the lobula. Scale bars, 5 μm. **b**, LPLC2 is a VPN cell type without axonal topography. LPLC2 axon terminals do not form a topographic map along the D–V axis of the lobula as visualized from EM reconstruction (left and middle) and light microscopy (right, *n* = 6 pairs of clones). Scale bars, 5 μm. **c**, Relationship between synaptic gradients and topography of axon terminals for different VPN types (see Extended Data Fig. 8 for more examples). *LC6 retains coarse axonal retinotopy^25^. **LC10 was previously shown to have A–P axonal retinotopy^6,13^. A, anterior; P, posterior; D, dorsal; M, medial; L, lateral.

**Extended Data Fig. 8:**
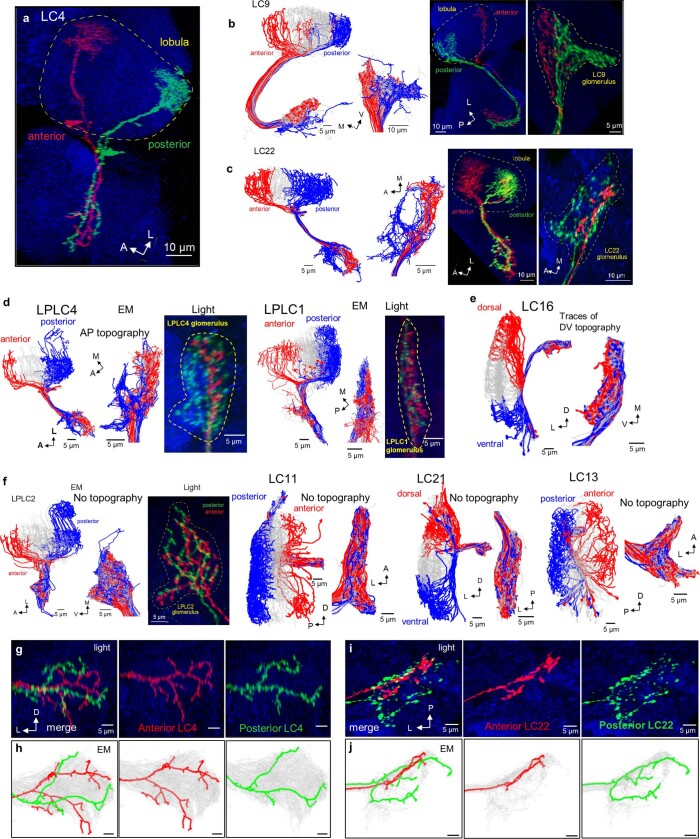
Topographic mapping in VPN optic glomeruli. **a**–**d**, Examples of VPN cell types with retinotopic mapping of axon terminals reflecting the AP axis of the lobula dendritic map. Assessed via EM reconstructions and light microscopy (individual cells are labeled using Multicolor Flp-Out). LC4 neurons (**a**, same pair as in Fig. 5a) with dendrites innervating anterior and posterior lobula have axon terminals in distinct regions of the glomerulus. LC9 (b), LC22 (c), LPLC4 and LPLC1 (d) show similar axonal topography. Left panels, hemibrain connectome reconstructions of 15 anterior (red), 15 posterior (blue), and the remaining cells (grey). Corresponding reconstruction of axons in the VPN glomeruli shows visual separation of anterior and posterior terminals. Right panels: one anterior (red) and one posterior (green) cell labeled using Multicolor Flp-Out and assessed via light microscopy. (n = 4 pairs of A-P individual clones from different brains with reproducible axon terminal topography for LPLC4 and LPLC1, n = 5 for LC9, n = 6 for LC22). **e**, Traces of DV axonal retinotopy in LC16 – a single example found across 20 VPN cell types. **f**, Representative examples of VPNs without topographic mapping of axon terminals in optic glomeruli (EM reconstructions), despite elaborating synaptic gradients reflecting visual space map (see Fig. 4h and Extended Data Fig. 7). **g**–**j**, Differential axon terminal morphology and glomerular targeting between individual LC4 (**g**, **h**) and LC22 neurons (**i**, **j**) innervating anterior and posterior regions of the lobula (N = 9 pairs of A-P individual clones from different brains with reproducible axon terminal topography for LC4, n =64 for LC22, correspond to examples from Fig. 5). Characteristic branching patterns are consistent between light microscopy and EM-based connectome reconstruction. A, anterior; P, posterior; D, dorsal; V, ventral; L, lateral; M, medial.

Topographic arrangement of VPN axons would provide a simple mechanism for the development of synaptic gradients. Previous studies concluded that this was unlikely^6,15,25,26^ (with an exception of LC10 (refs. ^6,13^) and traces of topography in the LC6 (ref. ^25^) glomerulus). Here we revisited this issue using EM data^27^ and looked for axon topography corresponding to dendritic arrangement along either the A–P or D–V axis of the lobula. We found five additional VPN cell types (LC4, LC9, LC22, LPLC1 and LPLC4) that have axon terminals retaining rough A–P topography, and one (LC16) whose axons maintain traces of D–V topography (Fig. 5a, Extended Data Fig. 8a–e and Supplementary Videos 3 and 4). These observations were confirmed using light microscopy and MultiColor FlpOut^40^: the axon terminals of sparsely labelled VPNs with dendrites in either the anterior or posterior lobula targeted distinct domains in their corresponding glomeruli and also exhibited differential morphology as assessed by EM and light microscopy (Extended Data Fig. 8g–j). No axon topography, however, was observed for most (12/20) VPN cell types (Fig. 5b and Extended Data Fig. 8f) at the resolution of our analysis. Therefore, synaptic gradients in these cases (Fig. 4h and Extended Data Fig. 7) must emerge by an alternative mechanism.

In summary, VPNs fall into two classes (Fig. 5c). In one, synaptic gradients correlate with axon topography within the glomerulus and in the other they do not.

## DN dendrite location matches LC4 synaptic gradients

**Fig. 6:**
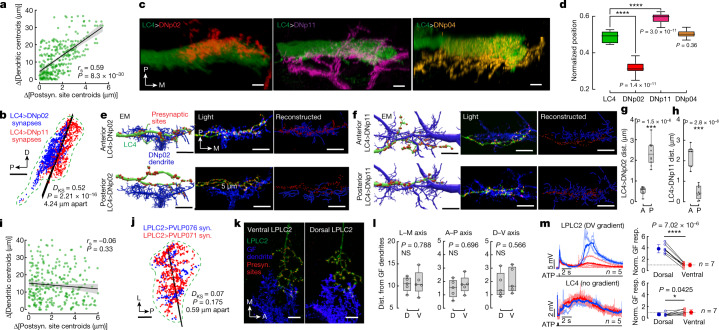
Synaptic gradients in VPNs emerge through spatial and spatially independent mechanisms. **a–h**, LC4: spatial mechanism. **a**, Relationship between dendritic map and spatial arrangement of synapses in the glomerulus for LC4 (for 300 pairs of top 25 postsynaptic (postsyn.) partners); *r*_s_, Spearman’s rank correlation coefficient. Error bands, s.e.m. **b**, Location of DNp02 and DNp11 postsynaptic sites in the retinotopic LC4 glomerulus (green dashed outline); black line, separation plane; *D*_KS_, two-tailed Kolmogorov–Smirnov test. Scale bar, 5 μm. **c**, Confocal projections of LC4 glomeruli and DN dendrites. Scale bars, 5 μm. **d**, Normalized DN dendritic centroid position within the LC4 glomerulus (*n* = 12 brains each; one-way ANOVA, Dunnett’s test, versus LC4 glomerulus centroid, ****P < 0.0001). **e**,**f**, Single anterior and posterior LC4 neurons with labelled presynaptic sites colocalized with DNp02 and DNp11 dendrites, alongside their EM reconstructions (bodyID 1907587934 and 1249932198). Scale bars, 5 μm. **g**,**h**, Distance (dist.) between dendrites of DNp02 (**g**) and DNp11 (**h**) and presynaptic sites in anterior versus posterior LC4 (*n* = 8 and 10 brains, respectively; two-tailed unpaired Welch’s *t*-test, ***P < 0.001). **i–m**, LPLC2: non-spatial mechanism. **i**, Relationship of input and output centroids (as in **a**) for LPLC2. **j**, Location of PVLP071 and PVLP076 postsynaptic sites in the LPLC2 glomerulus (statistics as in **a**,**b**), which lacks retinotopy. Scale bar, 5 μm. **k**, Single ventral and dorsal LPLC2 neurons with labelled presynaptic (presyn.) sites colocalized with the GF dendrite. Scale bars, 5 μm. **l**, Distances between presynaptic sites of single dorsal versus ventral LPLC2 neurons and GF dendrites, measured along three cardinal axes (*n* = 5 brains each; two-tailed unpaired Welch’s *t*-test, NS, *P *> 0.05). **m**, GF depolarization responses from localized activation of dorsal versus ventral LPLC2 and LC4 neurons expressing the P2X_2_ receptor. Left: representative GF responses (*n* = 5, one fly); individual (lighter-coloured lines) and averaged (darker lines) responses. Right: comparison of normalized average GF responses (resp.) to dorsal versus ventral VPN activation (two-tailed paired *t*-test; error bars, s.e.m., *P ≤ 0.05, ****P < 0.0001). Responses were averaged during the late response peak; see Extended Data Fig. 12c for quantification of the early peak. *n*, individual flies tested. A, anterior; P, posterior; D, dorsal; V, ventral; L, lateral; M, medial. All box plots show median and interquartile range. Source data

**Extended Data Fig. 9:**
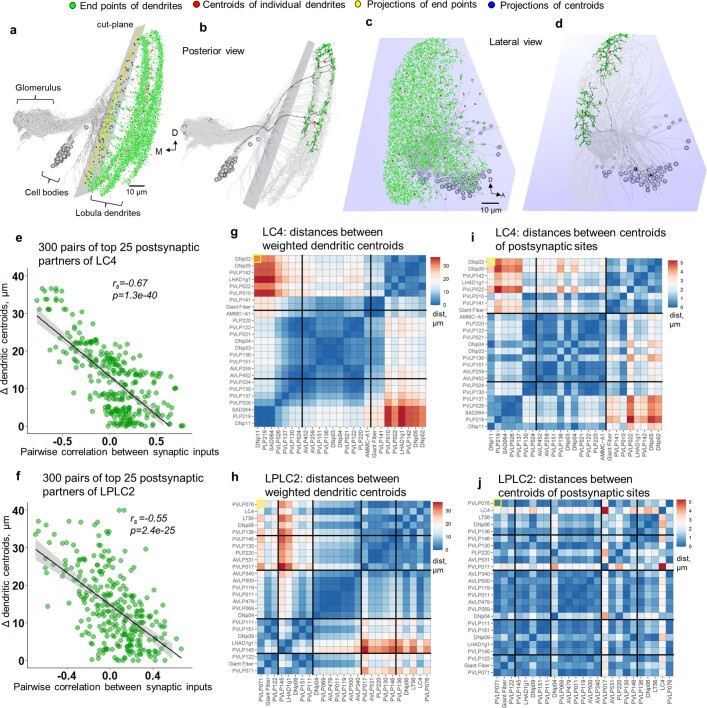
EM-based analysis of synaptic connectivity patterns in LC4 and LPLC2. **a**–**d**, Analysis of the spatial distribution of dendritic centroids in the lobula for LC4. Centers of mass (centroids) of dendrites for individual 71 LC4 neurons (red dots) were established based on coordinates of end branching points (green dots) located laterally from the separation cut-plane (grey) to exclude the branching points in the glomerulus. Branching points and centroids were projected onto the plane (yellow and blue dots, respectively). Shown for the entire LC4 population and two representative neurons from posterior (**a**–**b**) and lateral (**c**–**d**) views. Similar analysis was also done for LPLC2. D, dorsal; M, medial; A, anterior. **e**–**f**, Relationship between synaptic input specificity and spatial dendritic map (measured by positions of weighted dendritic centroids) for 300 pairs of top 25 postsynaptic partners of LC4 (**e**) and LPLC2 (**f**). r_s_, Spearman’s rank correlation coefficient. Error bands, SEM. Strong correlation indicates that LC4 and LPLC2 neurons with neighboring dendrites have similar synaptic specificity in the glomerulus. **g**–**h**, Matrices of pairwise distances between weighted centroids of inputs for 300 pairs of the top 25 postsynaptic partners of LC4 (**g**) and LPLC2 (**h**). The order of postsynaptic neurons is preserved from Fig. 4d and f. **i**–**j**, Matrices of pairwise distances between centroids of postsynaptic sites of the top 25 postsynaptic partners of LC4 (**i**) and LPLC2 (**j**). The order of postsynaptic neurons is preserved from Fig. 4d and f. Source data

We focused on LC4 to understand how axon topography leads to the formation of synaptic gradients. We found that for the top 25 postsynaptic partners of LC4, the spatial distribution of postsynaptic sites in the LC4 glomerulus strongly correlated with the positions of LC4 dendrites in the lobula (Fig. 6a, Extended Data Fig. 9g,i and Methods). This is exemplified by DNp02 and DNp11 receiving anticorrelated inputs from LC4 axons (Figs. 2h and 4d,e) and having spatially segregated postsynaptic sites in the LC4 glomerulus (Fig. 6b and Extended Data Fig. 9i). Topographic mapping of the LC4 axon terminals alone cannot account for these patterns.

**Extended Data Fig. 10:**
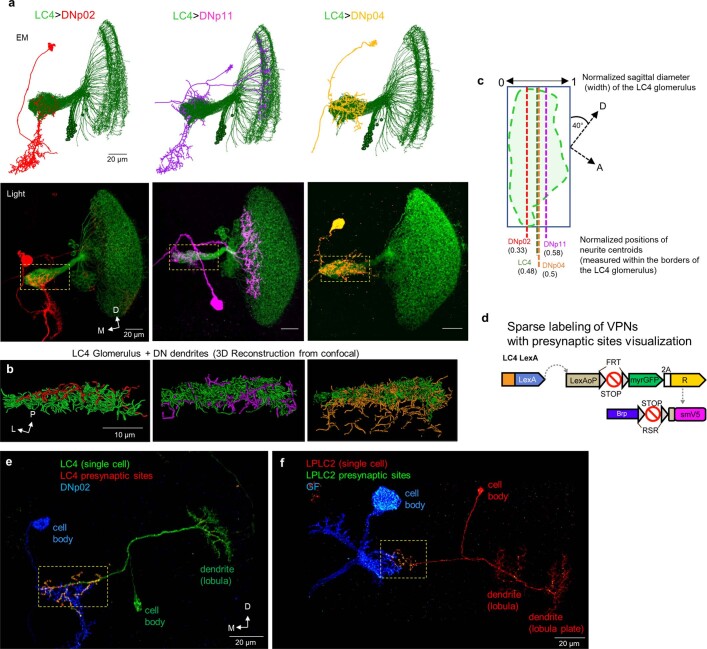
Assessment of wiring strategy in VPN glomeruli using light-level neuroanatomy. **a**, Top: EM-based connectome reconstructions of LC4 neurons (green) and three DNs. Bottom: confocal projections of colocalized LC4 and three DNs, LC4 glomerulus is indicated with a dashed yellow rectangle (n = 12 brains for each LC4-DN, corresponding to Fig. 5c,d). Note that DNp11 has an additional dendritic branch in the lobula. **b**, Imaris reconstructions of confocal image stacks: LC4 glomerulus (axons) and dendritic segments of three DNs (both reconstructed as filaments) as indicated. **c**, Assessment of spatial distribution of DN dendrites within the volume of the LC4 glomerulus (outlined with a green dashed line). Topographic separation of the LC4 axon terminals occurs along the sagittal diameter of the glomerulus. Normalized value of the sagittal diameter was used to assess the relative placement of the postsynaptic dendrites (see Methods). Dotted straight lines indicate the positions of DN dendritic centroids along the sagittal diameter of the glomerulus. Position of the LC4 glomerulus centroid slightly deviates from 0.5 value due to the naturally curved shape of the glomerulus. **d**, Strategy for sparse labeling of LC4 neurons and their presynaptic sites. Labeling of cell membranes (myr::GFP) and presynaptic sites (Brp-smGdP-V5) is dependent upon heat-shock induced expression of FLP (See Methods). **e**, Confocal projection of a single LC4 neuron with presynaptic sites labeled and colocalized with dendrite of DNp02 (n = 18 individual LC4 neurons from different brains, corresponding to Fig. 6e–h. **f**, Confocal projection of a single LPLC2 neuron with labeled presynaptic sites colocalized with GF dendrite (n = 10 individual LPLC2 neurons from different brains, corresponding to Fig. 6k,l). Regions corresponding to LC4 and LPLC2 glomeruli are indicated with dashed yellow rectangles.

To assess whether the spatial distribution of DN dendrites also contributes to differential connectivity, we mapped the positions of dendrites of different DN neurons within the LC4 glomerulus using light microscopy. DNp02 and DNp11 dendrites occupy unique glomerular sub-compartments where axons of LC4 corresponding to anterior and posterior visual fields selectively terminate. By contrast, dendrites of DNp04, a postsynaptic neuron with no A–P synaptic gradient with LC4, arborize uniformly within the LC4 glomerulus (Fig. 6c,d and Extended Data Fig. 10a–c).

To map synapses at the light level, we used a modification of the STaR^41^ method to visualize presynaptic sites in sparsely labelled LC4 neurons (Extended Data Fig. 10d,e) and assessed their proximity to DNp02 and DNp11 dendrites (Fig. 6e–h). The presynaptic sites of LC4 from the anterior lobula were much closer on average to the DNp02 dendrites than those from the posterior (Fig. 6e,g and Supplementary Videos 5 and 6). Conversely, DNp11 dendrites were closer to the presynaptic sites of LC4 from the posterior lobula.

In summary, LC4 utilizes a spatial wiring strategy to attain graded synaptic connectivity. A combination of topographic arrangement of LC4 axons and placement of DNp02 and DNp11 dendrites within different spatial domains in the glomerulus determines the directional specificity of the escape response to looming stimuli from different regions of the visual field.

## Spatially independent synaptic gradients in LPLC2

The synaptic gradients elaborated by LPLC2 form in a fundamentally different way from those elaborated by LC4. Analysis of the top 25 postsynaptic partners of LPLC2 found no significant relationship between positions of LPLC2 dendrites in the lobula (that is, synaptic output specificity) and the spatial arrangement of synapses in the LPLC2 glomerulus (Fig. 6i and Extended Data Fig. 9h,j). For example, the postsynaptic neurons PVLP071 and PVPL076 have anticorrelated inputs from LPLC2 (Fig. 4f,g), yet their postsynaptic sites are intermingled in the LPLC2 glomerulus (Fig. 6j and Extended Data Fig. 9j).

**Extended Data Fig. 11:**
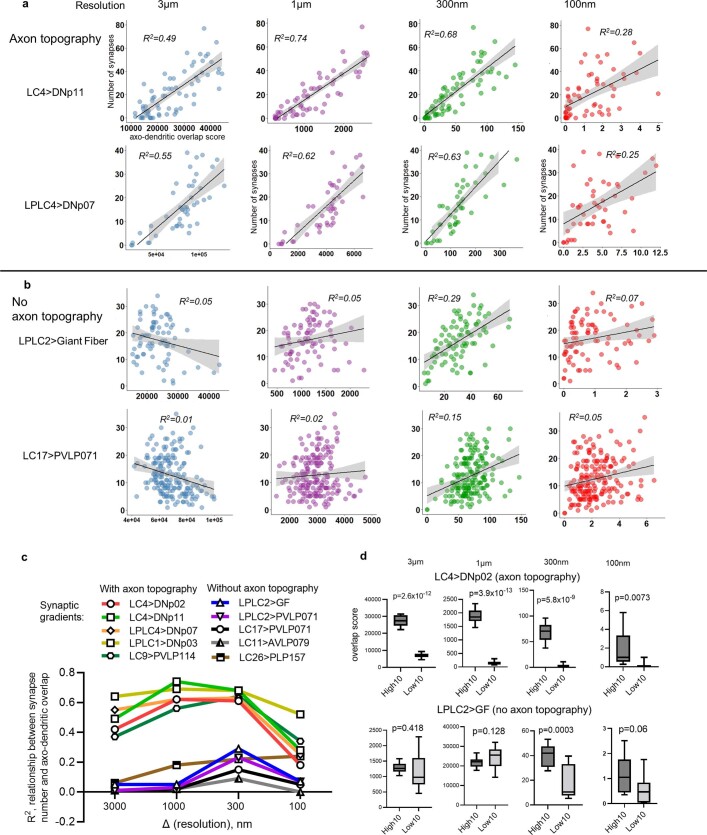
The role of axo-dendritic overlap in synaptic specificity with and without axon topography. Relationship between the number of synapses (VPNs and their postsynaptic targets) and axo-dendritic overlap score obtained from “hemibrain” EM volume using “overlap_score” function of the *natverse* package. **a**–**b**, representative examples of correlation plots featuring VPNs with (**a**) and without (**b**) topographic mapping in optic glomeruli (each dot represents a single neuron within one VPN cell type) and their individual postsynaptic partners. Measurements were performed using four resolution thresholds (3000, 1000, 300 and 100nm) to progressively distinguish the specific role of general axonal topography (retinotopic mapping of axonal projections) from microtopography resulting from local synaptic specificity. An R^2^ value reflects the impact of spatial positioning of axons and dendrites on the resulting synaptic connectivity. Error bands, SEM. D, dorsal; M, medial; P, posterior; L, lateral. **c**, plots summarizing the impact of topography on synaptic connectivity (at different spatial resolutions) for four VPN cell types with and without axonal topography and their five different postsynaptic partners (all examples are taken form Fig. 4 and Extended Data Fig. 7). **d**, Comparison of axo-dendritic overlap score for top10 individual VPN neurons with and without axonal topography (LC4, top and LPLC2, bottom, respectively, n = 10+10 for each VPN cell type) making most and least number of synapses with their postsynaptic targets (DNp02 and GF, respectively). Two-tailed unpaired Welch’s *t*-test. In all box plots, the solid line depicts the median; the upper and lower bounds of the box depict the third and first quantiles of the data spread, respectively. Whiskers indicate min and max values. Source data

We confirmed this principle by labelling presynaptic sites in axons of individual LPLC2 neurons with dendrites within the dorsal and ventral lobula and measuring the proximity of these presynaptic sites to the GF dendrites (Fig. 6k and Extended Data Fig. 10f). No significant difference in distances was found (Fig. 6l) despite a marked difference in synapse counts (Fig. 4h). Thus, the spatial distribution of synapses in the LPLC2 glomerulus seems random. To assess this principle in a more systematic manner, we further analysed EM data (hemibrain) and measured the correlation between axo-dendritic overlap and synaptic counts for four topographic and four non-topographic VPNs and their postsynaptic partners (Extended Data Fig. 11). Our results strengthened the notion that VPNs utilize two qualitatively different wiring strategies to form synaptic gradients.

**Extended Data Fig. 12:**
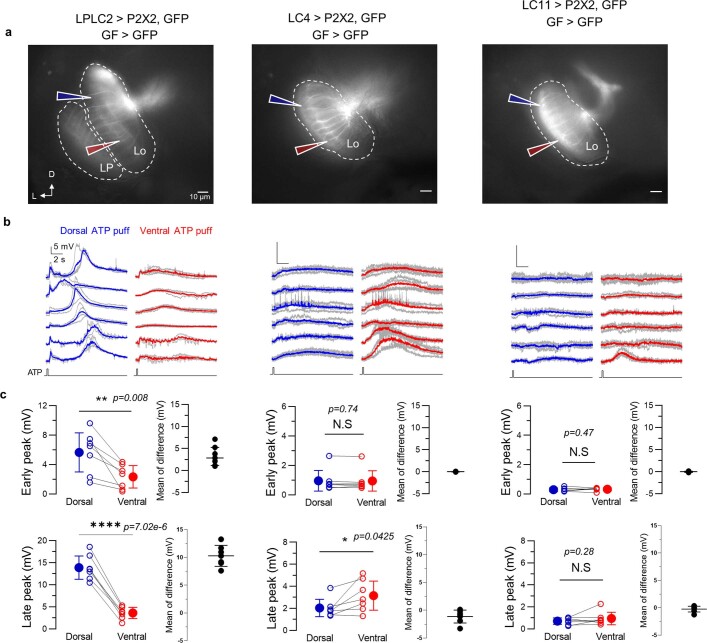
Functional assessment of synaptic gradients in LPLC2 using electrophysiology. **a**, Single focal plane snapshots of GFP expression by three tested VPN cell types overlaid with schematic ATP puffing locations. Focal application of 5 mM ATP (0.2 s pulse) was performed at either dorsal or ventral position along the dendrites of each VPN in the lobula. Outlines of the lobula (Lo) and lobula plate (LP), dashed white lines. D, dorsal; L, lateral. **b**, Depolarization responses of GF upon activation of VPNs from 6 different flies in each genotype (data from one animal shown in Fig. 6m). Blue, dorsal averaged responses. Red, ventral averaged responses. Grey, individual trials. **c**, Summary of early (top) and late (bottom) GF peak responses obtained in the time window of 0.5s and 8s after the stimulation onset, respectively. Mean differences between absolute values of GF responses to dorsal and ventral stimulations of the corresponding VPN cell types are shown next to each plot. Early peak: 3.32 ± 2.25 mV for LPLC2, 0.007 ± 0.06 mV for LC4, −0.04 ± 0.13 mV for LC11. Late peaks: 10.27 ± 1.88 mV for LPLC2, −1.12 ± 1.16 mV for LC4, −0.23 ± 0.52 mV for LC11 (error bars, SEM; n = 7 animals for each genotype. Individual data points are means of n = 5 trials per animal; two-tailed paired t-test). N.S.: P >0.05; *: P <0.05; **: P <0.01; ****: P <0.0001. Detailed description of statistical tests and p-values for panels is available in Supplementary Table 1. Source data

We next sought to assess whether the synaptic gradients of LPLC2 onto the GF were functionally significant (Fig. 4h). The dendrites of LPLC2 neurons expressing the P2X_2_ receptor were locally activated by injection of ATP in the dorsal and ventral regions of the lobula, and the response in the GF was assessed using electrophysiological recordings (Extended Data Fig. 12a). GF responses following activation of dorsal LPLC2 were significantly stronger than those following ventral ATP injections. By contrast, little difference was seen in response following stimulation of dorsal versus ventral LC4 (also connected to the GF, but without a notable D–V synaptic gradient; Fig. 6m and Extended Data Fig. 12b,c).

In summary, functionally relevant graded synaptic connectivity of LPLC2 is established through a spatially independent mechanism.

## Discussion

We took advantage of cell-type-specific genetic tools, behavioural and physiological analyses, and densely reconstructed neuron connectivity maps to examine a central brain sensory-to-motor interface at synaptic resolution. We showed that the transformation of object location from retinal to body coordinates is solved by gradients of synapses between spatially ordered visual-feature-detecting neurons (that is, VPNs) and movement-direction-specific premotor neurons (that is, DNs). We demonstrated that such numeric gradients produce functional synaptic weights and lead to predictable response differences in postsynaptic neurons that drive fly escape takeoffs correctly oriented away from looming threats. Individual cells within one VPN cell type are thus functionally heterogeneous with connectivity profiles often as dissimilar as ones found between different neuron types. It is this continuous heterogeneity that converts visual stimuli into ethologically relevant behavioural responses.

We discovered behavioural roles for individual DNs (DNp02 and DNp11), and it may be tempting to consider these as command neurons for particular body movement directions. However, several observations suggest that they act instead as members of a larger DN group whose combined activity represents both the strength of the drive to takeoff and movement direction. First, when optogenetically activated alone no LC4-DN drove a high takeoff rate (25% takeoff rate maximum, all long-mode takeoffs). By contrast, activation of the command-like GF drove nearly 100% takeoff (all short-mode takeoffs). Second, activation of DN combinations (for example, DNp02 and DNp04 or DNp02, DNp04 and DNp06), increased takeoff rates significantly, although only up to about 40% takeoff. This suggests that co-activation of multiple DNs drives the long-mode takeoff and more DNs than we identified probably participate. Finally, whereas co-activation of DNp02 and DNp04 increased the backward shift of flies compared to activation of DNp02 alone, this shift was reduced by additional co-activation of DNp06. Thus, different DNs may ‘vote’ for movement in a particular direction and the resulting behaviour is the sum of these votes, much like the population activity in directionally selective motor cortex neurons correlates with movement direction in primates^42^. This mechanism could extend beyond forward and backward control if the left and right DNs of the same type, which would be differentially activated in the event of a looming stimulus from the side, also independently ‘voted’ for leftward or rightward body shifts, much like unilateral activity in DNg02 neurons correlates with left or right flight saccades in flying flies^43^. By this mechanism it would be plausible for the fly to obtain the ability to takeoff in any direction relative to its body, as has been observed in behavioural data.

Expanding our analysis to 20 different VPN cell types and their postsynaptic partners revealed synaptic gradients as a general property of visual feature detector output in *Drosophila*. Evidence consistent with a gradient motif has been observed at the sensorimotor interface of the cockroach cercal system, where input from directionally selective abdominal wind-sensitive hairs has graded effects on the response of downstream giant interneurons, which drive escape^44^. Thus, synaptic number gradients may be a general principle for transmission of spatial information between sensory and motor networks.

VPNs guide innate visual behaviours of the fly, including looming-evoked backing or takeoff and small-object tracking^6,16^. We expect that the synaptic gradients we described here are specified by genetically hard-wired developmental processes, rather than through experience. In support of a developmental origin, we observed substantially the same LC4-DN gradients in EM volumes of two different fly brains^27,38^. The same wiring motif, however, could be present in more flexible areas of sensorimotor interface such as ellipsoid body ‘compass’ neurons^45^ and would provide a simple mechanism for how learning-induced changes in numbers of synapses between neurons could result in different stimulus–behaviour pairings.

We identified two different circuit wiring strategies producing synaptic gradients in different VPN cell types. In the ‘spatial’ strategy, topographic mapping of VPN axon terminals organizes the optic glomerulus and is ‘read out’ by stereotypically positioned dendrites of different target neurons. Axonal topography may arise through age-dependent mechanisms as described for more peripheral regions of the fly visual system^46^, or through graded expression of cell surface molecules (for example, Eph receptors and ephrins) as described in the vertebrate visual system^47^. Developmental mechanisms must act in parallel to target dendritic processes of different postsynaptic neuron types to discrete domains within the glomerulus.

Most VPN cell types we examined (12/20), however, did not show clear topographic organization of their axonal projections. Thus, in most cases, gradients emerge in the absence of spatial cues. Molecular heterogeneity within one cell type previously found in the fly visual system^48^ and mouse visual cortex^49^ may underlie such differential synaptic specificity. Future work should examine whether spatial gradients of molecular regulators instruct differential expression of cell adhesion and recognition molecules in VPNs, thereby transforming a retinotopic arrangement of dendritic arbours in the optic lobe into a graded distribution of synapses in the central brain.

## Methods

### Experimental model details

Flies were reared under standard conditions at 25 °C and 50% humidity with a 16-h light/8-h dark cycle on a standard cornmeal fly food. Male and female flies 3–5 days after eclosion were used for all experiments except if specified otherwise. Flies used for optogenetic activation experiments were raised on 0.2 mM retinal (Sigma R2500) food, and maintained on 0.4 mM retinal food as adults. These flies were kept in the dark in foil-covered vials until they were prepared for experiments. Supplementary Table 2 provides detailed descriptions of fly genotypes used in each experiment and origins of transgenic stocks.

### Behavioural experiments

#### High-throughput takeoff assay

We tested escape responses of unrestrained flies using our previously developed FlyPEZ^33^ system to automate fly behaviour experiments and collect large sample sizes necessary to quantitatively characterize differences in escape behaviour. In FlyPEZ, individual flies were released one at a time onto a 5 mm by 5 mm glass platform through an automated gate without undue perturbation, where they were targeted for visual or optogenetic stimulation. The fly position on the platform was tracked using a real-time tracking algorithm, which coordinated the triggering of a high-speed video camera and either looming stimulus or light stimulus. For visual stimulation, we used digital micromirror device projectors running at a refresh rate of 360 Hz, controlled by MATLAB using the Psychophysics Toolbox. Dark looming discs expanding from 10° to 180° at an elevation of 45° and azimuth of 0°, 90° or 180° ± 22.5° relative to the fly head position were presented on a 7-inch-diameter back-projection coated dome centred over the fly platform, which covers 360° in azimuth and 120° in elevation of the fly’s visual field. To simulate an object approaching with constant velocity, the projected looming disc centre remained constant while the disc radius increased nonlinearly over time on the basis of the following equation

$$\theta \left(t\right)=2{{\rm{\tan }}}^{-1}\frac{l}{{vt}}$$

in which $\theta$ is the angular size of the stimulus (in radians), *l* is the radius of the virtual object, and *v* is its simulated approach velocity. $t$ = 0 is the theoretical time of contact, when the object would reach 180°, so that *t* < 0 during object expansion. For optogenetic stimulation, CsChrimson was activated in flies raised on retinal food with four 624-nm wavelength light-emitting diodes (total irradiance of 500 W m^−2^, as measured from the location of the fly on the platform). Escape responses were captured using a macro lens on a high-speed camera, and two perspectives of the fly (side and bottom views) were filmed at 6,000 frames per second under 850-nm infrared illumination. Only one stimulus was presented per fly, and the platform was cleared before release of the subsequent fly. All looming experiments were carried out during the 4-h activity peak in the afternoon light cycle, and all optogenetic experiments were carried out in the dark.

#### Behavioural data analysis

Escape sequence durations in the CsChrimson activation and Kir2.1-silencing experiments were manually annotated by labelling the first frame of wing raising and the last frame of tarsal contact from the FlyPEZ video data. For the analysis of postural shifts and takeoff angles following either optogenetic activation or looming stimulus presentation, we used a machine learning software package, Animal Part Tracker (APT, a software package developed by the Branson Lab at Janelia) v0.3.4, which allowed us to automatically track locations of body parts in the input videos. For automated tracking, the videos were subsampled at 600 Hz (1.67-ms interval), which was sufficient to observe smooth changes in leg and body movements. Missing tracking data due to occlusions (body part out of frame) were interpolated for gaps less than five frames (8.33 ms), and a moving-average filter was applied to smooth the raw tracking data. For optogenetic activation experiments, videos in which visibility of T2 legs was lost over the 100 ms of annotation were excluded, except for cases in which the fly performed a takeoff. For silencing experiments, videos in which visibility of T2 legs was lost between the stimulus start and the start of jumping leg extension were excluded from the COM movement, COM flow field and T2 leg angle analyses. Individual takeoff vectors were obtained from two locations of the COM, one at takeoff, when the last of the middle tarsi loses contact with the ground (*t*_end_), and one either at a manually annotated frame of the start of jumping leg extension, or at 5 ms before the takeoff (*t*_start_; Fig. 1i). The population mean resultant length, $\bar{R}$, is calculated by the following equation

$$\bar{R}=\frac{1}{n}\left|\mathop{\sum }\limits_{j=0}^{n}{{\rm{e}}}^{i\theta j}\right|$$

in which $n$ is the total number of the takeoff vectors, and ${{\rm{e}}}^{i\theta }$ is Euler’s formula as a simplified representation of a vector. $\bar{R}$ is a statistic between 0 and 1 for the spread of a circular variable in the population, such that 1 means all of the takeoff directions are concentrated at a single angle, and 0 means the spread is more uniform. The COM referenced to fly body-centric coordinates was obtained by translating and rotating the COM as described in Extended Data Fig. 1c. Δ[T2 leg angle] at a given time frame of the FlyPEZ video was obtained using the APT-tracked tarsal tips of the middle legs and the COM as described in Fig. 1d. A Butterworth filter was applied to the T2 leg angle time series results. Individual COM movement vectors were calculated as the vector from COM_0_ to COM_pre_ (Extended Data Fig. 1d).

### Electrophysiological experiments

#### Electrophysiological recordings and data analysis

Female flies of 2–4 days in age were anaesthetized on a Peltier-driven cold plate and positioned ventral side up to be tethered on a custom polyether-ether-ketone recording plate by applying ultraviolet-cure glue to the head and thorax. We used only female flies because: female flies are larger and hence less prone to desiccation than male flies, and so have the potential to provide longer-lasting electrophysiological recordings; and both the hemibrain and full brain (FAFB) EM datasets were collected from female flies, so our direct measurements of the gradients are both in female flies. For recording stability, the proboscis was glued in a retracted position and the front pair of legs were clipped and glued at the femur. To access the DN soma for whole-cell recording, a window was cut in the cuticle on the posterior side of the head, and the overlying fat and trachea were removed. The brain was continuously perfused during electrophysiology with the external solution containing (in mM): 103 NaCl, 3 KCl, 5 *N*-Tris (hydroxymethyl)methyl-2-aminoethane-sulfonic acid, 8 trehalose, 10 glucose, 26 NaHCO_3_, 1 NaH_2_PO_4_, 1.5 CaCl_2_ and 4 MgCl_2_, bubbled with 95% O_2_ and 5% CO_2_, and adjusted to pH 7.3 and 273–276 mOsm. To disrupt the perineural sheath around the soma of interest, collagenase (0.25 mg ml^−1^ in external solution) was applied locally with a large-bore pipette to the surface of the brain. A small amount of tissue was then removed by using suction from a pipette filled with external solution to gain unrestricted patch pipette access. Patch pipettes were made from borosilicate glass using a Sutter p-1000 puller and fire-polished after pulling using a Narishige MF-900 microforge to achieve a final resistance of 4–8 MΩ. The internal solution contained (in mM): 140 potassium aspartate, 10 4-(2-hydroxyethyl)-1-piperazineethanesulfonic acid, 1 ethylene glycol tetraacetic acid, 4 MgATP, 0.5 Na_3_GTP and 1 KCl. The pH was 7.3 and the osmolarity was adjusted to approximately 265 mOsm. To obtain patch-clamp recordings, DN somata were visually targeted through brief GFP excitation. Recordings were acquired in current-clamp mode with a MultiClamp 700B amplifier (Molecular Devices), low-pass filtered at 10 kHz, and digitized at 40 kHz (Digidata 1440A, Molecular Devices).

Whole-cell recording data were analysed in MATLAB using custom written code or using Clampfit 11 software (Molecular Devices), and graphical representation was carried out by using Prism 9.2.0 software (GraphPad). Spike events in response to looming stimuli were determined on the basis of the rise slope (mV ms^−1^) in the response region above a threshold given from the averaged maximum slope in the baseline region across individual recordings, followed by visual inspection of the raw data. The baseline region of each trial corresponded to the 2-s time window before the beginning of the looming stimulus. The response region was the 150-ms period after the onset of the stimulus. To estimate the magnitude of depolarization in response to looming stimuli, membrane potentials were averaged across individual trials (4–8 trials per neuron), and the area (ms × mV) was calculated in the 150-ms response region.

#### Visual stimulation for electrophysiology

Custom visual stimuli were produced in MATLAB using the Psychophysics Toolbox to display looming stimuli with different approach angles around the fly. We were limited in how far posterior we could show stimuli owing to constraints of the plate to which the fly was tethered to for accessing the back of the head capsule and the microscope. This was especially an issue for DNp11 recordings, as the microscope objective blocks presentation of the posterior stimuli that should most strongly excite DNp11. Thus, our strategy for assessing the functional gradient of the receptive field (RF) was to compare directly measured visual responses in the experimentally accessible visual field to responses predicted by a model we generated from the measured synaptic numbers and an alignment with the visual world (see the section below entitled Mapping the LC4 anatomical RF). Within our accessible visual area, we generated looming stimuli at 32.5°, 45°, 57.5° and 70° along the eye equator (anterior to posterior) and then pitched the plane of these stimuli down 20° to roughly coincide with the tilt of the synaptic gradients we measured. Looming stimuli from different azimuths were shown in randomized sets. Looming stimuli were arrays of three discs, black on a white background, and programmed to expand from 0° to 30° in azimuth in each disc with a 12-s inter-stimulus interval. We used three-disc vertical arrays because we wanted to use a stimulus that would produce as strong a response as possible and which could be varied in azimuth. As LC4 neurons have only an approximately 40° RF, only a handful of LC4 neurons may be excited by a single looming stimulus. Therefore, to activate more LC4 neurons along a given azimuth, we used a column of three. See Extended Data Fig. 4a for a depiction of the looming stimuli used. Visual stimuli were back-projected at 360 Hz onto a 4-inch diameter dome at 768 × 768 resolution. Stimulus frames were synchronized by simultaneously recording a photodiode with the recording trace that monitored a patch of each frame projected just outside the dome and coloured black or white on alternate frames. Constant angular velocity stimuli were generated using the following equation

$$\theta \left(t\right)={v}_{{\rm{a}}}t$$

in which $\theta$ is the angular size of the stimulus, ${v}_{{\rm{a}}}$ is the angular velocity, and $\theta$ = 0 at $t$ = 0. All stimuli were corrected for distortion and irradiance differences as described previously.

#### P2X_2_ experiments

Whole-cell patch-clamp recordings from the GF were carried out in 2–4-day-old female flies as described above. For P2X_2_ receptor activation of LC4 or LPLC2 VPNs, a glass capillary pulled to a 1-μm diameter was positioned on the VPN dendrites, which expressed both GFP and the P2X_2_ receptor, approximately 50 μm below the surface of the brain. ATP (Sigma A9187, 5 mM) was microinjected (5 psi, 200-ms pulse) under the control of a Picospritzer (Parker Hannifin). To test dorsoventral gradients of functional connectivity between the VPNs and the GF, either the dorsal or ventral part of the lobula was stimulated in an alternating fashion at 90-s intervals to permit recovery between pulses. Whole-cell recording data were analysed as mentioned above. Before calculating the peak amplitudes of the GF response, the membrane potential traces acquired during ATP applications were low-pass filtered and averaged across individual trials as specified in the figure legends.

### Generation of single-cell STaR transgenic flies

A combination of HIFI DNA assembly (NEB) and restriction-enzyme-based cloning was used to generate either 13XLexAoP2-FRT-STOP-FRT-myr::GFP-2A-R::PEST or 13XLexAoP2-FRT-STOP-FRT-myr::tdTomato-2A-R::PEST through modification of pJFRC177 (Addgene: 10XUAS-FRT-STOP-FRT-myrGFP, plasmid no. 32149). First, the 10XUAS sequence of pJFRC177 was replaced by 13XLexAoP2 from pJFRC19 (Addgene: 13XLexAoP2-IVS-myrGFP, plasmid no. 26224). Second, the GFP-coding sequence of pJFRC177 was replaced by either GFP-2A (cassette C: GS linker-FRT-STOP-FRT-GFP-2A-LexAVP16) or tdTomato-2A (UAS-DIPalpha-2A-tdTomato), both followed by the coding sequence of R::PEST recombinase from pJFRC165 (Addgene: 20XUAS-IVS-R::PEST plasmid no. 32142). Transgenic flies were generated by integration of either construct into the VK00033 landing site using a commercial injection service (BestGene). To generate sparsely labelled VPNs with visualized presynaptic sites (sparse StaR), 13XLexAoP2-FRT-STOP-FRT-myr::GFP-2A-R::PEST constructs were recombined with StaR^41^ (Brp-RSRT-stop-RSRT-myr::smGdP-V5-2A-LexA, laboratory stock). Female flies carrying the recombined constructs were crossed into male flies with VPN-specific LexA driver lines and hsFLP recombinase. At 48 h after puparium formation, pupae were heat-shocked for 15 min in 37 °C water bath.

### Immunohistochemistry

Unless otherwise specified, dissected flies were aged 3–4 days post eclosion. Brains were dissected in ice-cold Schneider’s *Drosophila* Medium (Gibco 21720–024), and fixed in acid-free glyoxal (Addax Biosciences) containing 5% sucrose (Sigma S9378) overnight at 4 °C. Brains were rinsed repeatedly with PBST (PBS (Bioland Scientific LLC PBS01-03) containing 0.5% Triton-X100 (Sigma T9284)), and incubated in blocking solution (PBST containing 10% normal goat serum (Sigma G6767)) for 2 h at room temperature before incubation with antibodies. Brains were incubated sequentially with primary and secondary antibodies diluted in blocking solution for 24 h at 4 °C, with three rinses in PBST followed by 1 h incubations at room temperature in between and afterwards. Primary antibodies were used at 1:20 (nc82), 1:500 (chicken anti-GFP) and 1:200 (all others) dilutions. All secondary antibodies were used at 1:300 dilutions. The full list of antibodies used is available in the Reporting Summary. The technique for subsequent mounting in DPX was adapted from the Janelia protocol for mounting the central nervous system of adult *Drosophila* in DPX. After being washed to remove residual secondary antibodies, brains were additionally fixed with PBS containing 4% paraformaldehyde (Electron Microscopy Sciences 15710) for 3 h at room temperature, rinsed with PBS and mounted on 22 × 22-mm square No. 1.5H cover glass (Thorlabs CG15CH2) (with the posterior side of the brain facing the cover glass) previously coated with poly-l-lysine (0.078% solution in deionized water, Sigma P1524) with added 0.2% Kodak Photo-Flo 200 Solution (Electron Microscopy Sciences 74257) followed by a quick 1–2-s rinse with MilliQ water. Brains were dehydrated by placing the cover glass into baths with successively increasing ethanol (Sigma 459844) concentrations (30–50–75–95–100–100–100%, 10 min each) followed by three successive baths of xylene (Thermo Fisher Scientific X5–500), 5 min each. Afterwards the glass was uniformly covered with 8–10 drops of DPX (Electron Microscopy Sciences 13510) and placed on a prepared slide between the spacers made of two 22 × 22 mm square No. 2 cover glasses (Fisher Scientific 12-540B). The slide was left for 24 h in the hood for drying, and then transferred to room temperature and imaged at least 24 h afterwards,

### Confocal image acquisition and processing

Immunofluorescence images were acquired using a Zeiss LSM 880 confocal microscope with Zen digital imaging software using an oil-immersion ×63 objective. Serial optical sections were obtained from whole-mount brains with a typical resolution of 1,024 μm × 1,024 μm × 0.5 μm. Image stacks were exported to Imaris 9.7 for level adjustment, cropping and removal of signal in off-target brain regions and background noise, as well as 3D volume reconstructions.

### Analysis of neuroanatomical data from confocal image stacks

To assess and measure the differential placement of DN dendrites within the LC4 glomerulus, confocal image stacks of colocalized glomeruli and DN dendrites were aligned so that the *x* axis corresponded to the sagittal diameter (width) of the glomerulus and cropped at the edges of the glomerulus to exclude any extraglomerular DN dendrites from consideration. 3D reconstructions of LC4 axon terminals and DN dendrites were obtained using the Imaris Filaments tool (Extended Data Fig. 10b). The *x* coordinates of the filaments were exported to GraphPad Prism 9.2.0 and normalized to the sagittal diameter of the LC4 glomerulus (0–1 range). The *x* coordinate of the centroid of the DN dendritic arbour was calculated as a mean of *x* coordinates of all filaments and used as a final metric of spatial distribution of dendrites within the glomerulus (Extended Data Fig. 10c).

To assess the spatial proximity between presynaptic sites of individual LC4 or LPLC2 neurons and DN dendrites (single-cell STaR experiments), Brp puncta in single VPN cells were reconstructed using the Imaris Spots tool, followed by identification of their centroids, as well as centroids of reconstructed dendritic filaments. Distance between Brp puncta and DN dendrite centroids was measured along the sagittal diameter of the glomerulus (LC4) or along three cardinal axes (A–P, D–V and L–M) of the glomerulus (for LPLC2). Only female flies were used for analysis to be consistent with the available connectome data, which are in a female fly. Analyser was not blinded to genotype due to characteristic identifiable morphology of DNp02, DNp11 and DNp04, as well as clear anatomical positions of anterior–posterior LC4 and LPLC2.

### Connectomics analysis

#### FAFB connectome reconstruction analysis

We annotated the FAFB serial section transmission EM volume using the CATMAID software to determine the chemical synaptic connectivity between the LC4 neurons and four DNs of interest, DNp02, DNp11, GF and DNp04. As a starting point, we used previously traced skeletons for LC4 neurons. To start tracing the DNs, we used morphological cues from confocal fluorescence imaging in distinct strategies to locate a starting point for tracing each DN. For DNp02, confocal microscopy stacks suggested that the somata neurite travels close to the path of the GF somata neurite. We found DNp02 by locating its neurite within a shared soma tract, which, along with several other neurites, appears encased in a dark sheath. DNp04 was located when tracing the LC4 neurons. The skeleton was then traced out and linked to the same soma tract as DNp02 and GF. DNp11 was located by searching for candidate DNs that cross the midline dorsal of the oesophagus. From each starting node, the full skeleton was traced and compared to the confocal image stacks for confirmation of cell type identity. To determine the chemical synaptic connectivity, we searched for four criteria: T-bars, presynaptic vesicles, synaptic clefts and postsynaptic densities. If a potential synapse possessed two out of four criteria, it was labelled as a synapse. We focused our efforts on LC4 (presynaptic) and DNp02, DNp11, GF and DNp04 (postsynaptic) synapses to gain a representative view of the connectivity between LC4 and the DNs.

#### Mapping the LC4 anatomical RF

To model the real-world RFs of the LC4 population, we followed a previously established method^25^, and applied it to newly reconstructed LC4 neurons. We first mapped all 55 LC4 dendrites (FAFB volume) onto a layer of the lobula by fitting a second-order surface to all of the dendritic arbours. Each projected dendrite traced out a polygon that represented the field of view of the corresponding LC4 neuron. We modelled each LC4 as a 2D circular Gaussian on this surface. Its height was set to be unity, and its width was given by the radius of a circle that had the same area as the projected polygon. To map each LC4 neuron’s location (COM of the dendrite) onto eye coordinates, we used as reference points previously reconstructed Tm5 neurons^25^ from two medulla columns, which correspond to the centre of the eye and a dorsal position on the central meridian (the line that partitions the eye between anterior and posterior halves). To estimate an LC4-DN’s RF, we first multiplied each LC4 Gaussian’s height by the number of synaptic connections to that LC4-DN. We then summed all LC4 Gaussians to produce a 2D multi-Gaussian distribution, which was the LC4-DN’s RF. To estimate an LC4-DN’s response to a looming stimulus, we multiplied the LC4 Gaussian’s height by both the number of synaptic connections and the percentage of the LC4 RF that was covered by the stimulus at its maximum size (30°). For instance, if the stimulus overlapped with 40% of an LC4‘s RF, then that LC4 Gaussian’s effective height was the number of connections times 0.4. Finally, all LC4 contributions were summed to produce the estimated response of the LC4-DN to the looming stimulus. Note that LC4s that did not overlap at all with a stimulus contributed nothing to the DN’s response.

#### Hemibrain connectome reconstruction analysis

Volumetric data of neurons and neuropils, as well as connectivity data and synapse locations, were obtained from the neuPrint (hemibrain v1.1) database, (https://neuprint.janelia.org/) and have been processed with the natverse package^51^ for R (v4.0.3) using custom scripts. All coordinates in these datasets are based on the original voxel resolution of 8 nm.

##### ***k***-means clustering of individual neurons within VPN cell type populations

For each VPN cell type, a matrix of synaptic connections between individual VPN neurons and their postsynaptic partners was constructed using the neuprintR package. Postsynaptic partners forming fewer than 50 total synapses with the entire VPN cell type population were excluded (about 1 synapse per individual VPN on average; we reasoned that this threshold would reflect the limit of EM data reconstruction error rate). Synaptic connections within the population of VPN cell type were also removed (for example, LC4 to LC4 synapses). The resulting matrix was scaled such that the variables (individual postsynaptic partners) had unit variance across the observations (individual VPN cells in the population). Principal component analysis was carried out on the scaled matrix. Up to ten principal components were used for *k*-means clustering on the individual VPNs (the number of PCs was determined on the basis of the drop in the eigenvalues in the scree plots for each VPN type). A value of *k* was subsequently determined from the corresponding scree plots by the drop in the within-cluster sum of squared distance (example in Extended Data Fig. 6a).

##### Correlation in synaptic connectivity

Matrices of correlation in synaptic connectivity (Fig. 4d,f) were generated using the pairwise Spearman’s correlation coefficient of the 300 unique pairs derived from the top 25 postsynaptic partners (based on the total number of synapses and excluding connections with the same VPN cell type) of LC4 and LPLC2, ordered using hierarchical clustering. Each entry evaluates the monotonic relationship between a pair of the synaptic connectivity gradients. For each pair, the correlation coefficient was calculated using the vectors containing the number of synapses between the selected postsynaptic partners and each individual VPN cell within the population (example in Fig. 2h).

##### Weighted cendritic centroids

To evaluate the distances between weighted dendritic map centroids for each postsynaptic partner of LC4 and LPLC2, we identified the endpoints of the dendrites innervating the lobula for each individual VPN cell. These were isolated using cut-planes that were manually selected to optimally separate the lobula region (Extended Data Fig. 9a–d). We then evaluated the centroid of the selected endpoints by calculating their spatial average. We repeated these steps for all VPN cells within a population (71 for LC4 and 85 for LPLC2). The resulting 3D centroids were then projected onto the cut-plane. The outlines of the lobula were obtained by evaluating the convex hull of the projections of all the selected endpoints for all of the cells of the examined VPN. To identify a weighted innervation centroid for a given postsynaptic partner, we calculated the overall weighted median using the number of synapses associated with each centroid as weights. We then identified the top anticorrelated pairs of postsynaptic partners by selecting those for which the Spearman’s correlation coefficient is below a certain threshold that was determined by evaluating, for each VPN, the value that optimizes the correlation between the dendritic map and the synaptic connectivity correlation. For each one of these top pairs, we estimated the perpendicular to the line connecting the corresponding weighted median centroids. These lines were combined using the median operator to reduce the influence of potential outliers. This resulted in a single line identifying the optimal unbiased separator of the most anticorrelated pairs (median separation line in Fig. 4e,g). The distance between their projections onto the line perpendicular to the optimal separator (projection line in Fig. 4e,g) was used as a final metric to generate the matrix and calculated for each pair of postsynaptic partners (Extended Data Fig. 9g,h). The projection line for LC4 was almost parallel to the A–P axis of the lobula (Fig. 4e), and slightly deviated from that for LPLC2 owing to the dual nature of synaptic gradients in this cell type (both A–P and D–V).

##### Spatial distribution of postsynaptic sites in optic glomeruli

A similar approach based on the estimation of an unbiased separator was used to evaluate the correlation between the centroids of postsynaptic sites for postsynaptic partners of VPNs. To estimate this separator, we started by isolating all postsynaptic sites within the glomerulus using a cut-plane. We then selected the top anticorrelated pairs of postsynaptic partners, in a manner similar to how we analysed the dendritic map centroids. For each pair, we split the postsynaptic sites into two different classes depending on the postsynaptic partner they belonged to and used a support vector machine with a linear kernel to evaluate the optimal separating plane. We then computed the median of these planes. This resulted in a single plane identifying the unbiased optimal separator of the most anticorrelated pairs (median separation plane in Fig. 6b,j). We then projected the postsynaptic sites of each postsynaptic partner onto the line perpendicular to the optimal separator and calculated the distance between the median of the respective projections. The distance matrices for a given VPN cell type were obtained by calculating the pairwise distances between each of the 300 pairs of postsynaptic partners of LC4 and LPLC2 (Extended Data Fig. 9i,j). For selected pairs of postsynaptic neurons, the distributions of postsynaptic sites projections were compared using the two-sample Kolmogorov–Smirnov test (Fig. 6b,j).

##### Assessment of topographic mapping in VPN optic glomeruli

Skeletons of individual neurons within each VPN cell type were selected manually on the basis of A–P and D–V topographic location of their dendrites and/or the pattern of *k*-means clustering of the dendritic maps (15 cells per topographic domain, unless stated otherwise in the figure legends). Groups of neurons with dendrites in different topographic domains were differentially coloured. Axonal processes of the corresponding neurons were traced in the optic glomerulus and visually examined for traces of spatially ordered organization. LC10 neurons were excluded from the analysis owing to previously reported A–P axonal topography^6,13^. LC6 neurons were excluded owing to previous extensive analysis^25^ indicating the presence of coarse glomerular retinotopy inaccessible through visual examination.

### Statistical analysis

All statistical analyses were carried out in RStudio 1.4.1103, MATLAB or Prism 9.2.0 software (GraphPad). NS: *P *> 0.05, *P < 0.05, **P < 0.01, ***P < 0.001 and ****P < 0.0001 for all figures where applicable. Statistical tests for Figs. 1e and 3e,h and Extended Data Figs. 1,2,4 and 12 are described in Supplementary Table 1. In all box plots (Fig. 6 and Extended Data Fig. 11), the solid line depicts the median; the upper and lower bounds of the box depict the third and first quantiles of the data spread, respectively. Whiskers indicate minimum and maximum values. All other statistical tests, number of replicates, statistical significance levels and other elements of statistical analysis are reported in the corresponding section of the Methods, along with the associated results and/or in the corresponding figure legends. No data were excluded from the analysis except as noted for the behaviour experiments (see the section in the Methods entitled Behavioural data analysis). All measurements were taken from distinct samples.

### Reporting summary

Further information on research design is available in the Nature Portfolio Reporting Summary linked to this article.

## Online content

Any methods, additional references, Nature Portfolio reporting summaries, source data, extended data, supplementary information, acknowledgements, peer review information; details of author contributions and competing interests; and statements of data and code availability are available at 10.1038/s41586-022-05562-8.

## Supplementary information

Supplementary Information This file contains Supplementary Tables 1 and 2 and legends for Supplementary Videos 1–6.

Reporting Summary

Supplementary Video 1

Supplementary Video 2

Supplementary Video 3

Supplementary Video 4

Supplementary Video 5

Supplementary Video 6

## Data Availability

The datasets generated during the current study are available as downloadable files at https://data.mendeley.com/datasets/pnbmx825wv, https://data.mendeley.com/datasets/84kh3ncbf8 and https://data.mendeley.com/datasets/th99hk824v. These include confocal image stacks related to Figs. 4–6. Other datasets generated and/or analysed during the current study are available from the corresponding authors on reasonable request. For further information regarding any resources and reagents, contact G.M.C. (gwyneth.card@columbia.edu) or S.L.Z. (lzipursky@mednet.ucla.edu). Source data are provided with this paper.

## Source data

Source Data Figs. 1–4 and 6 and Extended Data Figs. 1–4, 9, 11 and 12

## Code availability

R scripts for hemibrain connectome reconstruction analysis are available at https://github.com/avaccari/DrosophilaVPNWiring. Code associated with APT v0.3.4 (developed by the Branson Lab at Janelia) is available at https://zenodo.org/record/6366082. All reconstructed neurons from the FAFB dataset will be made available at https://fafb.catmaid.virtualflybrain.org/. All code to carry out the analysis and generate the figures on the FAFB data will be available at https://github.com/artxz/LC4_code.

## References

[1] Crochet S, Lee SH, Petersen CCH (2019). Neural circuits for goal-directed sensorimotor transformations. Trends Neurosci..

[2] Calhoun AJ, Murthy M (2017). Quantifying behavior to solve sensorimotor transformations: advances from worms and flies. Curr. Opin. Neurobiol..

[3] Cavanaugh J (2012). Optogenetic inactivation modifies monkey visuomotor behavior. Neuron.

[4] Bianco IH, Engert F (2015). Visuomotor transformations underlying hunting behavior in zebrafish. Curr. Biol..

[5] Buneo CA, Jarvis MR, Batista AP, Andersen RA (2002). Direct visuomotor transformations for reaching. Nature.

[6] Wu M (2016). Visual projection neurons in the *Drosophila* lobula link feature detection to distinct behavioral programs. Elife.

[7] Otsuna H, Ito K (2006). Systematic analysis of the visual projection neurons of *Drosophila melanogaster*. I. Lobula-specific pathways. J. Comp. Neurol..

[8] Coen P, Xie M, Clemens J, Murthy M (2016). Sensorimotor transformations underlying variability in song intensity during *Drosophila* courtship. Neuron.

[9] Huston SJ, Jayaraman V (2011). Studying sensorimotor integration in insects. Curr. Opin. Neurobiol..

[10] Helmbrecht TO (2018). Topography of a visuomotor transformation. Neuron.

[11] Heukamp AS, Warwick RA, Rivlin-Etzion M (2020). Topographic variations in retinal encoding of visual space. Annu. Rev. Vis. Sci..

[12] Klier EM, Wang H, Crawford JD (2001). The superior colliculus encodes gaze commands in retinal coordinates. Nat. Neurosci..

[13] Timaeus L, Geid L, Sancer G, Wernet MF, Hummel T (2020). Parallel visual pathways with topographic versus nontopographic organization connect the *Drosophila* eyes to the central brain. iScience.

[14] Aptekar JW, Keleş MF, Lu PM, Zolotova NM, Frye MA (2015). Neurons forming optic glomeruli compute figure–ground discriminations in *Drosophila*. J. Neurosci..

[15] Keleş MF, Frye MA (2017). Object-detecting neurons in *Drosophila*. Curr. Biol..

[16] Klapoetke NC (2022). A functionally ordered visual feature map in the *Drosophila* brain. Neuron.

[17] Städele C, Keleş MF, Mongeau JM, Frye MA (2020). Non-canonical receptive field properties and neuromodulation of feature-detecting neurons in flies. Curr. Biol..

[18] Bidaye SS (2020). Two brain pathways initiate distinct forward walking programs in *Drosophila*. Neuron.

[19] Ribeiro IMA (2018). Visual projection neurons mediating directed courtship in *Drosophila*. Cell.

[20] Sen R (2017). Moonwalker descending neurons mediate visually evoked retreat in *Drosophila*. Curr. Biol..

[21] Zacarias R, Namiki S, Card GM, Vasconcelos ML, Moita MA (2018). Speed dependent descending control of freezing behavior in *Drosophila*
*melanogaster*. Nat. Commun..

[22] Ache JM, Namiki S, Lee A, Branson K, Card GM (2019). State-dependent decoupling of sensory and motor circuits underlies behavioral flexibility in *Drosophila*. Nat. Neurosci..

[23] Cande J (2018). Optogenetic dissection of descending behavioral control in *Drosophila*. Elife.

[24] Namiki S, Dickinson MH, Wong AM, Korff W, Card GM (2018). The functional organization of descending sensory-motor pathways in *Drosophila*. Elife.

[25] Morimoto MM (2020). Spatial readout of visual looming in the central brain of *Drosophila*. Elife.

[26] Panser K (2016). Automatic segmentation of *Drosophila* neural compartments using GAL4 expression data reveals novel visual pathways. Curr. Biol..

[27] Scheffer LK (2020). A connectome and analysis of the adult *Drosophila* central brain. Elife.

[28] Card G, Dickinson MH (2008). Visually mediated motor planning in the escape response of *Drosophila*. Curr. Biol..

[29] Tanaka R, Clark DA (2022). Neural mechanisms to exploit positional geometry for collision avoidance. Curr. Biol..

[30] Muijres FT, Elzinga MJ, Melis JM, Dickinson MH (2014). Flies evade looming targets by executing rapid visually directed banked turns. Science.

[31] Fotowat H, Gabbiani F (2011). Collision detection as a model for sensory-motor integration. Annu. Rev. Neurosci..

[32] Eaton, R. *Neural Mechanisms of Startle Behavior* (Springer, 1984).

[33] Williamson R, Peek MY, Breads P, Coop B, Card GM (2018). Tools for rapid high-resolution behavioral phenotyping of automatically isolated *Drosophila*. Cell Rep..

[34] Ache JM (2019). Neural basis for looming size and velocity encoding in the *Drosophila* giant fiber escape pathway. Curr. Biol..

[35] von Reyn CR (2017). Feature integration drives probabilistic behavior in the *Drosophila* escape response. Neuron.

[36] Von Reyn CR (2014). A spike-timing mechanism for action selection. Nat. Neurosci..

[37] Card GM (2012). Escape behaviors in insects. Curr. Opin. Neurobiol..

[38] Zheng Z (2018). A complete electron microscopy volume of the brain of adult *Drosophila melanogaster*. Cell.

[39] Klapoetke NC (2017). Ultra-selective looming detection from radial motion opponency. Nature.

[40] Nern A, Pfeiffer BD, Rubin GM (2015). Optimized tools for multicolor stochastic labeling reveal diverse stereotyped cell arrangements in the fly visual system. Proc. Natl Acad. Sci. USA.

[41] Chen Y (2014). Cell-type-specific labeling of synapses in vivo through synaptic tagging with recombination. Neuron.

[42] Georgopoulos AP, Kettner RE, Schwartzb AB (1988). Primate motor cortex and free arm movements to visual targets in three-dimensional space. II. Coding of the direction of movement by a neuronal population. J. Neurosci..

[43] Namiki S (2022). A population of descending neurons that regulates the flight motor of *Drosophila*. Curr. Biol..

[44] Hamon A, Guillet JC, Callec JJ (1990). A gradient of synaptic efficacy and its presynaptic basis in the cercal system of the cockroach. J. Comp. Physiol. A..

[45] Fisher YE, Lu J, D’Alessandro I, Wilson RI (2019). Sensorimotor experience remaps visual input to a heading-direction network. Nature.

[46] Pinto-Teixeira F (2018). Development of concurrent retinotopic maps in the fly motion detection circuit. Cell.

[47] Flanagan JG (2006). Neural map specification by gradients. Curr. Opin. Neurobiol..

[48] Kurmangaliyev YZ, Yoo J, Valdes-Aleman J, Sanfilippo P, Zipursky SL (2020). Transcriptional programs of circuit assembly in the *Drosophila* visual system. Neuron.

[49] Cheng S (2022). Vision-dependent specification of cell types and function in the developing cortex. Cell.

[50] Shinomiya K (2019). The organization of the second optic chiasm of the *Drosophila* optic lobe. Front. Neural Circuits.

[51] Bates AS (2020). The natverse, a versatile toolbox for combining and analysing neuroanatomical data. Elife.

