# Publisher Correction: Synaptic gradients transform object location to action

**DOI:** 10.1038/s41586-023-05930-y

**Published:** 2023-03-13

**Authors:** Mark Dombrovski, Martin Y. Peek, Jin-Yong Park, Andrea Vaccari, Marissa Sumathipala, Carmen Morrow, Patrick Breads, Arthur Zhao, Yerbol Z. Kurmangaliyev, Piero Sanfilippo, Aadil Rehan, Jason Polsky, Shada Alghailani, Emily Tenshaw, Shigehiro Namiki, S. Lawrence Zipursky, Gwyneth M. Card

**Affiliations:** 1 Department of Biological Chemistry, Howard Hughes Medical Institute, David Geffen School of Medicine, University of California, Los Angeles, Los Angeles, CA USA; 2 Janelia Research Campus, Howard Hughes Medical Institute, Ashburn, VA USA; 3 Department of Computer Science, Middlebury College, Middlebury, VT USA; 4 Present Address: Research Center for Advanced Science and Technology, University of Tokyo, Tokyo, Japan; 5 Present Address: Department of Neuroscience, Howard Hughes Medical Institute, The Mortimer B. Zuckerman Mind Brain Behavior Institute, Columbia University, New York, NY USA

**Keywords:** Neural circuits, Sensorimotor processing

Correction to: *Nature* 10.1038/s41586-022-05562-8 Published online 4 January 2023

In the version of this article originally published, the *x*-axis labels for Figure 3e,f,i and j incorrectly read “Disc size (°)” and have been corrected to read “Azimuth (°)” in the HTML and PDF versions of the article.

