# Supplementary material for: Synaptic gradients transform object location to action
Source: Nature. 2023 Jan 4;613(7944):534–42. doi: 10.1038/s41586-022-05562-8 (PMC9849133; doi:10.1038/s41586-022-05562-8)
Supplement: Supplementary file 1 — This file contains Supplementary Tables 1 and 2 and legends for Supplementary Videos 1–6. [file 41586_2022_5562_MOESM1_ESM.pdf]

nature portfolio

https://doi.org/10.1038/s41586-022-05562-8

Supplementary information

Synaptic gradients transform object location to action

In the format provided by the authors and unedited

Supplementary Information

Supplementary Table 1: additional information about statistical tests

Supplementary Table 2: list of full Drosophila genotypes used in each Figure

Supplementary Videos

Supplementary Video 1: Optogenetic activation of DNp11xCsChrimson flies.

Supplementary Video 2: Optogenetic activation of DNp02xCsChrimson flies.

Supplementary Video 3: Axon terminals of single LC4 neurons with dendrites in anterior (red) and posterior (green) lobula also occupy distinct spatial domains in the LC4 optic glomerulus.

Supplementary Video 4: Axon terminals of single LC9 neurons with dendrites in anterior (red) and posterior (green) lobula also occupy distinct spatial domains in the LC9 optic glomerulus.

Supplementary Video 5: Presynaptic sites (red) of a single LC4 neuron with dendrites in the anterior lobula colocalized with the dendrites of DNp02 (blue). Significant overlap is observed. Anterior LC4 typically form >45 synapses with DNp02

Supplementary Video 6: Presynaptic sites (red) of a single LC4 neuron with dendrites in the posterior lobula colocalized with the dendrites of DNp02 (blue). No significant overlap is observed. Posterior LC4 typically form <5 synapses with DNp02.

Supplementary Table 1

Figure 1e – statistical tests and p-values

Dunnett's multiple comparisons test	Summary	P Value
Empty vs. DNp11	****	<e-15
Empty vs. DNp02	****	<e-15
Empty vs. DNp02, DNp04	****	<e-15
Empty vs. DNp02, DNp04, DNp06	****	<e-15
Empty vs. DNp01	ns	0.992973895263672
Empty vs. DNp04	ns	0.999808465576172
Empty vs. DNp03	ns	0.999564373779297
Empty vs. DNp05	ns	0.625951367187500
Empty vs. DNp06	ns	0.103961059570312

Figures 3e, 3f – statistical tests and p-values

Fig.3e_DNp02

Dunnett's multiple comparisons test	Summary	P Value
azi_32.5 vs. azi_45	ns	0.480472656250000
azi_32.5 vs. azi_57.5	ns	0.210202014160156
azi_32.5 vs. azi_70	**	0.008429632568359

Fig.3e_DNp11

Dunnett's multiple comparisons test	Summary	P Value
azi_32.5 vs. azi_45	ns	0.655974658203125
azi_32.5 vs. azi_57.5	ns	0.512326635742187
azi_32.5 vs. azi_70	ns	0.361355859375000

Fig.3f_DNp02

Dunnett's multiple comparisons test	Summary	P Value
azi_32.5 vs. azi_45	ns	0.070642529296875
azi_32.5 vs. azi_57.5	**	0.003612633514404
azi_32.5 vs. azi_70	***	0.000796996498108

Fig.3f_DNp11

Dunnett's multiple comparisons test	Summary	P Value
azi_32.5 vs. azi_45	ns	0.784611035156250
azi_32.5 vs. azi_57.5	**	0.001566457748413
azi_32.5 vs. azi_70	**	0.003273193359375

Extended Data Figures 1e, 1h and 2f

The p-value (Bonf.) indicates the application of the post-hoc Bonferroni correction for multiple comparisons. ∗ for p < 0.05, ∗∗ < 0.01, ∗∗∗ < 0.001 and ∗∗∗∗ < 0.0001 .

Normal Approximation to the Binomial Z-test for Extended Data Fig. 1e							
Group 1	Group 2	$N_{Group\ 1}$	$N_{Group\ 2}$	Z-Score	p-value	p-value (Bonf.)	Significance Marker
empty (SS01062)	DL_CTRL	600	771	2.15E+00	3.18E-02	5.72E-01	NS
empty (SS01062)	GF (SS00727)	600	292	2.73E+01	0.00E+00	0.00E+00	∗∗∗∗
empty (SS01062)	GF (SS27721)	600	404	2.60E+01	0.00E+00	0.00E+00	∗∗∗∗
empty (SS01062)	DNp02 (SS01053)	600	327	8.31E-02	9.34E-01	1.68E+01	NS
empty (SS01062)	DNp02 (SS01554)	600	313	1.24E+00	2.16E-01	3.88E+00	NS
empty (SS01062)	DNp03 (SS01081)	600	325	7.42E-01	4.58E-01	8.24E+00	NS
empty (SS01062)	DNp03 (SS01596)	600	285	9.98E-01	3.19E-01	5.73E+00	NS
empty (SS01062)	DNp04 (SS00934)	600	276	4.63E+00	3.57E-06	6.43E-05	∗∗∗∗
empty (SS01062)	DNp04 (SS01080)	600	301	4.24E+00	2.27E-05	4.08E-04	∗∗∗
empty (SS01062)	DNp05 (SS02612)	600	311	4.59E-01	6.47E-01	1.16E+01	NS
empty (SS01062)	DNp05 (SS00865)	600	319	6.63E-02	9.47E-01	1.70E+01	NS
empty (SS01062)	DNp06 (SS02256)	600	318	1.22E+00	2.22E-01	4.00E+00	NS
empty (SS01062)	DNp11 (SS49010)	600	305	9.90E+00	0.00E+00	0.00E+00	∗∗∗∗
empty (SS01062)	DNp11 (SS49024)	600	385	1.16E+01	0.00E+00	0.00E+00	∗∗∗∗
empty (SS01062)	DNp11 (SS49051)	600	361	3.86E+00	1.15E-04	2.06E-03	∗∗
empty (SS01062)	DNp02, DNp04 (SS01544)	600	307	1.38E+01	0.00E+00	0.00E+00	∗∗∗∗
empty (SS01062)	DNp02, DNp04, DNp06 (SS02292)	600	281	1.35E+01	0.00E+00	0.00E+00	∗∗∗∗

Normal Approximation to the Binomial Z-test for Extended Data Fig. 1h							
Group 1	Group 2	$N_{Group\ 1}$	$N_{Group\ 2}$	Z-Score	p-value	p-value (Bonf.)	Significance Marker
DNp01	DNp04	620	70	1.74E+01	0.00E+00	0.00E+00	∗∗∗∗
DNp01	DNp11	620	227	2.42E+01	0.00E+00	0.00E+00	∗∗∗∗
DNp01	DNp02, DNp04	620	122	1.69E+01	0.00E+00	0.00E+00	∗∗∗∗
DNp01	DNp02, DNp04, DNp06	620	111	1.30E+01	0.00E+00	0.00E+00	∗∗∗∗

Normal Approximation to the Binomial Z-test for Extended Data Fig. 2f (l/V = 10 ms, azimuth = 90°)							
Group 1	Group 2	$N_{Group\ 1}$	$N_{Group\ 2}$	Z-Score	p-value	p-value (Bonf.)	Significance Marker
empty (SS01062)	DL_CTRL	137	240	1.58E+00	1.14E-01	2.40E+00	NS
empty (SS01062)	L1, L2 (SS00797)	137	172	4.73E+00	2.25E-06	4.72E-05	****
empty (SS01062)	GF (SS00727)	137	131	1.28E+00	2.02E-01	4.23E+00	NS
empty (SS01062)	GF (SS27721)	137	102	1.35E+00	1.77E-01	3.73E+00	NS
empty (SS01062)	DNp02 (SS01053)	137	107	1.54E+00	1.22E-01	2.57E+00	NS
empty (SS01062)	DNp02 (SS01554)	137	143	7.86E-01	4.32E-01	9.07E+00	NS
empty (SS01062)	DNp03 (SS01081)	137	89	1.69E+00	9.02E-02	1.89E+00	NS
empty (SS01062)	DNp03 (SS01596)	137	122	8.00E-01	4.24E-01	8.90E+00	NS
empty (SS01062)	DNp04 (SS00934)	137	179	3.20E-01	7.49E-01	1.57E+01	NS
empty (SS01062)	DNp04 (SS01080)	137	122	1.57E+00	1.17E-01	2.46E+00	NS
empty (SS01062)	DNp05 (SS02612)	137	102	2.13E-01	8.32E-01	1.75E+01	NS
empty (SS01062)	DNp05 (SS00865)	137	113	8.19E-01	4.13E-01	8.66E+00	NS
empty (SS01062)	DNp06 (SS02256)	137	147	2.40E+00	1.62E-02	3.40E-01	NS
empty (SS01062)	DNp11 (SS49010)	137	109	1.27E-01	8.99E-01	1.89E+01	NS
empty (SS01062)	DNp11 (SS49024)	137	121	4.58E-01	6.47E-01	1.36E+01	NS
empty (SS01062)	DNp11 (SS49051)	137	116	1.21E-01	9.04E-01	1.90E+01	NS
empty (SS01062)	DNp02, DNp04 (SS01544)	137	130	1.43E+00	1.54E-01	3.23E+00	NS
empty (SS01062)	DNp02, DNp04, DNp06 (SS02292)	137	242	4.37E-01	6.62E-01	1.39E+01	NS

Normal Approximation to the Binomial Z-test for Extended Data Fig. 2f (l/V = 20 ms, azimuth = 90°)							
Group 1	Group 2	$N_{Group\ 1}$	$N_{Group\ 2}$	Z-Score	p-value	p-value (Bonf.)	Significance Marker
empty (SS01062)	DL_CTRL	107	263	9.66E-03	9.92E-01	2.08E+01	NS
ESC (SS01062)	L1, L2 (SS00797)	107	172	7.09E+00	1.37E-12	2.88E-11	****
empty (SS01062)	GF (SS00727)	107	132	2.05E+00	4.00E-02	8.41E-01	NS
empty (SS01062)	GF (SS27721)	107	104	4.45E-01	6.56E-01	1.38E+01	NS
empty	DNp02	107	120	1.73E+00	8.39E-02	1.76E+00	NS

(SS01062)	(SS01053)						
empty (SS01062)	DNp02 (SS01554)	107	136	1.29E+00	1.98E-01	4.15E+00	NS
empty (SS01062)	DNp03 (SS01081)	107	108	3.69E-01	7.12E-01	1.50E+01	NS
empty (SS01062)	DNp03 (SS01596)	107	115	8.10E-01	4.18E-01	8.78E+00	NS
empty (SS01062)	DNp04 (SS00934)	107	118	2.53E+00	1.13E-02	2.37E-01	NS
empty (SS01062)	DNp04 (SS01080)	107	89	1.24E+00	2.14E-01	4.50E+00	NS
empty (SS01062)	DNp05 (SS02612)	107	83	2.15E-01	8.30E-01	1.74E+01	NS
empty (SS01062)	DNp05 (SS00865)	107	110	7.19E-01	4.72E-01	9.91E+00	NS
empty (SS01062)	DNp06 (SS02256)	107	164	1.91E+00	5.67E-02	1.19E+00	NS
empty (SS01062)	DNp11 (SS49010)	107	112	1.50E+00	1.34E-01	2.82E+00	NS
empty (SS01062)	DNp11 (SS49024)	107	127	4.14E-01	6.79E-01	1.43E+01	NS
empty (SS01062)	DNp11 (SS49051)	107	115	8.07E-01	4.20E-01	8.81E+00	NS
empty (SS01062)	DNp02, DNp04 (SS01544)	107	129	2.79E+00	5.28E-03	1.11E-01	NS
empty (SS01062)	DNp02, DNp04, DNp06 (SS02292)	107	252	2.37E+00	1.79E-02	3.77E-01	NS

Normal Approximation to the Binomial Z-test for Extended Data Fig. 2f (l/V = 40 ms, azimuth = 90°)							
Group 1	Group 2	$N_{Group 1}$	$N_{Group 2}$	Z-Score	p-value	p-value (Bonf.)	Significance Marker
empty (SS01062)	DL_CTRL	150	235	7.96E-01	4.26E-01	8.94E+00	NS
empty (SS01062)	L1, L2 (SS00797)	150	141	6.80E+00	1.08E-11	2.26E-10	****
empty (SS01062)	GF (SS00727)	150	119	2.46E-01	8.06E-01	1.69E+01	NS
empty (SS01062)	GF (SS27721)	150	95	8.66E-02	9.31E-01	1.96E+01	NS
empty (SS01062)	DNp02 (SS01053)	150	95	1.40E+00	1.61E-01	3.37E+00	NS
empty (SS01062)	DNp02 (SS01554)	150	143	2.24E+00	2.52E-02	5.29E-01	NS
empty (SS01062)	DNp03 (SS01081)	150	80	3.95E+00	7.74E-05	1.62E-03	**
empty (SS01062)	DNp03 (SS01596)	150	144	5.37E-01	5.91E-01	1.24E+01	NS
empty (SS01062)	DNp04 (SS00934)	150	122	1.21E+00	2.27E-01	4.78E+00	NS
empty (SS01062)	DNp04 (SS01080)	150	98	1.13E+00	2.57E-01	5.40E+00	NS
empty	DNp05	150	95	1.74E+00	8.20E-02	1.72E+00	NS

(SS01062)	(SS02612)						
empty (SS01062)	DNp05 (SS00865)	150	108	4.59E-01	6.46E-01	1.36E+01	NS
empty (SS01062)	DNp06 (SS02256)	150	119	1.66E+00	9.73E-02	2.04E+00	NS
empty (SS01062)	DNp11 (SS49010)	150	111	4.91E-01	6.24E-01	1.31E+01	NS
empty (SS01062)	DNp11 (SS49024)	150	111	1.10E+00	2.70E-01	5.66E+00	NS
empty (SS01062)	DNp11 (SS49051)	150	123	2.34E+00	1.92E-02	4.02E-01	NS
empty (SS01062)	DNp02, DNp04 (SS01544)	150	70	7.76E-01	4.38E-01	9.19E+00	NS
empty (SS01062)	DNp02, DNp04, DNp06 (SS02292)	150	232	1.18E+00	2.38E-01	5.00E+00	NS

Normal Approximation to the Binomial Z-test for Extended Data Fig. 2f (l/V = 80 ms, azimuth = 90°)							
Group 1	Group 2	$N_{Group\ 1}$	$N_{Group\ 2}$	Z-Score	p-value	p-value (Bonf.)	Significance Marker
empty (SS01062)	DL_CTRL	132	195	2.14E+00	3.26E-02	6.84E-01	NS
empty (SS01062)	L1, L2 (SS00797)	132	130	6.13E+00	8.52E-10	1.79E-08	****
empty (SS01062)	GF (SS00727)	132	104	7.15E-01	4.75E-01	9.97E+00	NS
empty (SS01062)	GF (SS27721)	132	106	3.20E-01	7.49E-01	1.57E+01	NS
empty (SS01062)	DNp02 (SS01053)	132	107	6.82E-01	4.95E-01	1.04E+01	NS
empty (SS01062)	DNp02 (SS01554)	132	108	1.18E+00	2.38E-01	4.99E+00	NS
empty (SS01062)	DNp03 (SS01081)	132	110	2.59E+00	9.52E-03	2.00E-01	NS
empty (SS01062)	DNp03 (SS01596)	132	112	7.50E-01	4.53E-01	9.52E+00	NS
empty (SS01062)	DNp04 (SS00934)	132	111	9.60E-01	3.37E-01	7.07E+00	NS
empty (SS01062)	DNp04 (SS01080)	132	111	1.38E+00	1.67E-01	3.52E+00	NS
empty (SS01062)	DNp05 (SS02612)	132	93	6.86E-01	4.93E-01	1.03E+01	NS
empty (SS01062)	DNp05 (SS00865)	132	93	1.80E+00	7.20E-02	1.51E+00	NS
empty (SS01062)	DNp06 (SS02256)	132	113	1.28E-01	8.98E-01	1.89E+01	NS
empty (SS01062)	DNp11 (SS49010)	132	102	1.80E+00	7.19E-02	1.51E+00	NS
empty (SS01062)	DNp11 (SS49024)	132	110	2.02E+00	4.32E-02	9.07E-01	NS
empty (SS01062)	DNp11 (SS49051)	132	104	1.49E+00	1.36E-01	2.86E+00	NS
empty	DNp02, DNp04	132	119	1.08E+00	2.81E-01	5.90E+00	NS

(SS01062)	(SS01544)						
empty (SS01062)	DNp02, DNp04, DNp06 (SS02292)	132	196	1.17E+00	2.41E-01	5.06E+00	NS

Normal Approximation to the Binomial Z-test for Extended Data Fig. 2f (l/V = 40 ms, azimuth = 0°)							
Group 1	Group 2	$N_{Group\ 1}$	$N_{Group\ 2}$	Z-Score	p-value	p-value (Bonf.)	Significance Marker
empty (SS01062)	DNp02 (SS01053)	417	237	3.32E+00	9.12E-04	4.56E-03	**
empty (SS01062)	DNp02 (SS01554)	417	232	1.88E+00	6.01E-02	3.00E-01	NS
empty (SS01062)	DNp11 (SS49010)	417	197	1.31E+00	1.91E-01	9.56E-01	NS
empty (SS01062)	DNp11 (SS49024)	417	221	7.61E-01	4.47E-01	2.23E+00	NS
empty (SS01062)	DNp11 (SS49051)	417	244	1.19E+00	2.33E-01	1.17E+00	NS

Normal Approximation to the Binomial Z-test for Extended Data Fig. 2f (l/V = 40 ms, azimuth = 180°)							
Group 1	Group 2	$N_{Group\ 1}$	$N_{Group\ 2}$	Z-Score	p-value	p-value (Bonf.)	Significance Marker
empty (SS01062)	DNp02 (SS01053)	459	230	8.93E-01	3.72E-01	1.86E+00	NS
empty (SS01062)	DNp02 (SS01554)	459	240	4.22E-02	9.66E-01	4.83E+00	NS
empty (SS01062)	DNp11 (SS49010)	459	215	3.45E-01	7.30E-01	3.65E+00	NS
empty (SS01062)	DNp11 (SS49024)	459	250	3.89E+00	1.01E-04	5.04E-04	***
empty (SS01062)	DNp11 (SS49051)	459	248	2.26E+00	2.39E-02	1.20E-01	NS

Extended Data Figures 4e and 4h

Extended Data Fig. 4f - Repeated-measures one-way ANOVA, Dunnett's test

Dunnett's multiple comparisons test	Summary	P Value
azi_32.5 vs. azi_45	ns	0.428603149414062
azi_32.5 vs. azi_57.5	ns	0.111100744628906
azi_32.5 vs. azi_70	ns	0.417252880859375

Extended Data Fig. 4h - Repeated-measures one-way ANOVA, Dunnett's test

GF

Dunnett's multiple comparisons test	Summary	P Value
azi_32.5 vs. azi_45	ns	0.485842675781250
azi_32.5 vs. azi_57.5	ns	0.564074096679688
azi_32.5 vs. azi_70	ns	0.983942498779297

DNp04

Dunnett's multiple comparisons test	Summary	P Value
azi_32.5 vs. azi_45	ns	0.174991772460938
azi_32.5 vs. azi_57.5	*	0.027667117309570
azi_32.5 vs. azi_70	*	0.015157412719727

Extended Data Fig. 12 – two-tailed paired t test.

LPLC2_Early peak

P value	0.008024420002466
P value summary	**
Significantly different (P < 0.05)?	Yes
One- or two-tailed P value?	Two-tailed

LC4_Early peak

P value	0.745984870468680
P value summary	ns
Significantly different (P < 0.05)?	No
One- or two-tailed P value?	Two-tailed

LC11_Early peak

P value	0.471832705383019
P value summary	ns
Significantly different (P < 0.05)?	No

One- or two-tailed P value?	Two-tailed

LPLC2_Late peak

P value	0.000007023231523
P value summary	****
Significantly different (P < 0.05)?	Yes
One- or two-tailed P value?	Two-tailed

LC4_Late peak

P value	0.042451931129013
P value summary	*
Significantly different (P < 0.05)?	Yes
One- or two-tailed P value?	Two-tailed

LC11_Late peak

P value	0.282560538153719
P value summary	ns
Significantly different (P < 0.05)?	No
One- or two-tailed P value?	Two-tailed

Supplementary Table 2

Fly stocks and sources

Strain	Identifier	Source
DL wild type	N/A	M.H. Dickinson, Caltech
UAS-CsChrimson-mVenus (attP18)	N/A	Klapoetke et al., 2014
pJFRC49-UAS-eGFPKir2.1 (attP2)	N/A	Von Reyn et al., 2014
pJFRC28-UAS-GFP (attP2)	N/A	Pfeiffer et al., 2012
Empty GAL4: R24A03-p65ADZp (attP40); R74C01-ZpGdbd (attP2)	SS01062	Namiki et al., 2018
L1, L2: R48H08_p65ADZp (attP40); R29G11_ZpGdbd (attP2)	SS00797	Tuthill et al., 2013
GF: R14A01_p65ADZp (attP40); R79H12_ZpGdbd (attP2)	SS00727	Namiki et al., 2018
GF: R17A04_p65ADZp (attP40); R68A06_ZpGdbd (attP2)	SS27721	Von Reyn et al., 2014
GF: R68A06-lexA(attP40)	N/A	BDSC# 52807
DNp02: VT063736_p65ADZp (attP40); R24A03_ZpGdbd (attP2)	SS01053	Namiki et al., 2018
DNp02: VT063736_p65ADZp (attP40); VT017647_ZpGdbd (attP2)	SS01554	Namiki et al., 2018
DNp03: R91C05_p65ADZp (attP40); R31B08_ZpGdbd (attP2)	SS01081	Namiki et al., 2018
DNp03: R29F12_p65ADZp (attP40); R37G07_ZpGdbd (attP2)	SS01596	Namiki et al., 2018
DNp04: VT032898_p65ADZp (attP40); VT048835_ZpGdbd (attP2)	SS00934	Namiki et al., 2018
DNp04: R84B12_p65ADZp (attP40); VT048835_ZpGdbd (attP2)	SS01080	Namiki et al., 2018
DNp05: VT047755_p65ADZp (attP40); VT003280_ZpGdbd (attP2)	SS02612	Namiki et al., 2018
DNp05: VT019060_p65ADZp (attP40); VT003280_ZpGdbd (attP2)	SS00865	Namiki et al., 2018
DNp06: VT019018_p65ADZp (attP40); VT017411_ZpGdbd (attP2)	SS02256	Namiki et al., 2018
DNp11: BJD110D01_p65ADZp (attP40); R18G08_ZpGdbd (attP2)	SS49010	Namiki et al., 2018
DNp11: BJD119F04_p65ADZp (attP40); R18G08_ZpGdbd (attP2)	SS49024	Namiki et al., 2018
DNp11: R18G08_p65ADZp (attP40); BJD100H09_ZpGdbd (attP2)	SS49051	Namiki et al., 2018
DNp11: VT025392_p65ADZp (attP40); VT057247_ZpGdbd (attP2)	SS02891	Namiki et al., 2018
DNp02, DNp04: VT048835_p65ADZp (attP40); VT017647_ZpGdbd (attP2)	SS01544	Namiki et al., 2018
DNp02, DNp04, DNp06: VT017411_p65ADZp (attP40); VT017647_ZpGdbd (attP2)	SS02292	Namiki et al., 2018
LC4: R47H04-p65ADZp (attP40); R72E01-ZpGdbd (attP2)	SS00315	Wu et al. 2016
LC4: R49C04-lexA(attP40)	N/A	BDSC# 53488
LC9: VT027704-p65ADZp (attP40); VT040569-ZpGdbd (attP2)	SS02645	Wu et al. 2016
LC22: R64G10-p65ADZp (attP40); R35B06-ZpGdbd (attP2)	OL0001B	Wu et al. 2016
LPLC1: R64G09-p65ADZp (attP40); R37H04-ZpGdbd (attP2)	OL0029B	Wu et al. 2016
LPLC2: R19G02-p65ADZp (attP40); R75G12-ZpGdbd (attP2)	OL0048B	Wu et al. 2016
LPLC2: R75G12-lexA(attP40)	N/A	BDSC# 54073
MCFO: hs-FLPG5.PEST;; 10xUAS(FRT.stop)myr::smGdP-V5-THS 10xUAS(FRT.stop)myr::smGdP-FLAG}, 10xUAS(FRT.stop)myr::smGdP-HA	N/A	BDSC# 64085
UAS-P2X2, UAS-GFP	N/A	von Reyn et al., 2014
Sparse StaR: LexAop-(FRT.stop)-myr::GFP-2A-R, brp-(RSRT.stop)-myr::smGdP-V5-2A-LexA	N/A	this paper
Sparse StaR: LexAop-(FRT.stop)-myr::tdTomato-2A-R, brp-(RSRT.stop)-myr::smGdP-V5-2A-LeA	N/A	this paper
10xUAS-IVS-myr::smGdP-HA(attP18)	N/A	BDSC# 62145
10xUAS-IVS-myr::smGdP-HA(attP18), 13xLexAop2-IVS-myr::smGdP-V5(attP8)	N/A	BDSC# 76358
LC11: R22H02-p65ADZp (attP40); R20G06-ZpGdbd (attP2)	OL0015B	Wu et al. 2016

Genotypes by figure

Stock/genotype	Cells/effector	Used in figure
20XUAS-CsChrimson-mVenus/w^+; +/+; +/+	DL wild type > CsChrimson	Ext Fig. 1
20XUAS-CsChrimson-mVenus/w^+; R24A03_p65ADZp/+; R74C01_ZpGdbd/+	Empty (SS01062) > CsChrimson	Fig. 1e-i, Ext Fig. 1
20XUAS-CsChrimson-mVenus/w^+; R14A01_p65ADZp/+; R79H12_ZpGdbd/+	GF (SS00727) > CsChrimson	Fig. 1e-i, Ext Fig. 1
20XUAS-CsChrimson-mVenus/w^+; R17A04_p65ADZp/+; R68A06_ZpGdbd/+	GF (SS27721) > CsChrimson	Fig. 1e-i, Ext Fig. 1
20XUAS-CsChrimson-mVenus/w^+; VT063736_p65ADZp/+; R24A03_ZpGdbd/+	DNp02 (SS01053) > CsChrimson	Fig. 1e-i, Ext Fig. 1
20XUAS-CsChrimson-mVenus/w^+; VT063736_p65ADZp/+; VT017647_ZpGdbd/+	DNp02 (SS01554) > CsChrimson	Fig. 1e-i, Ext Fig. 1

20XUAS-CsChrimson-mVenus/w^+; R91C05_p65ADZp/+; R31B08_ZpGdbd/+	DNp03 (SS01081) > CsChrimson	Ext Fig. 1
20XUAS-CsChrimson-mVenus/w^+; R29F12_p65ADZp/+; R37G07_ZpGdbd/+	DNp03 (SS01596) > CsChrimson	Ext Fig. 1
20XUAS-CsChrimson-mVenus/w^+; VT032898_p65ADZp/+; VT048835_ZpGdbd/+	DNp04 (SS00934) > CsChrimson	Fig. 1e-i, Ext Fig. 1
20XUAS-CsChrimson-mVenus/w^+; R84B12_p65ADZp/+; VT048835_ZpGdbd/+	DNp04 (SS01080) > CsChrimson	Fig. 1e-i, Ext Fig. 1
20XUAS-CsChrimson-mVenus/w^+; VT047755_p65ADZp/+; VT003280_ZpGdbd/+	DNp05 (SS02612) > CsChrimson	Ext Fig. 1

20XUAS-CsChrimson-mVenus/w^+; VT019060_p65ADZp/+; VT003280_ZpGdbd/+	DNp05 (SS00865) > CsChrimson	Ext Fig. 1
20XUAS-CsChrimson-mVenus/w^+; VT019018_p65ADZp/+; VT017411_ZpGdbd/+	DNp06 (SS02256) > CsChrimson	Ext Fig. 1
20XUAS-CsChrimson-mVenus/w^+; BJD110D01_p65ADZp/+; R18G08_ZpGdbd/+	DNp11 (SS49010) > CsChrimson	Fig. 1e-i, Ext Fig. 1
20XUAS-CsChrimson-mVenus/w^+; BJD119F04_p65ADZp/+; R18G08_ZpGdbd/+	DNp11 (SS49024) > CsChrimson	Fig. 1e-i, Ext Fig. 1
20XUAS-CsChrimson-mVenus/w^+; R18G08_p65ADZp/+; BJD100H09_ZpGdbd/+	DNp11 (SS49051) > CsChrimson	Fig. 1e-i, Ext Fig. 1
20XUAS-CsChrimson-mVenus/w^+; VT048835_p65ADZp/+; VT017647_ZpGdbd/+	DNp02, DNp04 (SS01544) > CsChrimson	Fig. 1e-i, Ext Fig. 1
20XUAS-CsChrimson-mVenus/w^+; VT017411_p65ADZp/+; VT017647_ZpGdbd/+	DNp02, DNp04, DNp06 (SS02292) > CsChrimson	Ext Fig. 1
w^+; +/+; pJFRC49-10XUAS-IVS-eGFPKir2.1/+	DL wild type > Kir2.1	Ext Fig. 2
w^+; R24A03_p65ADZp/+; R74C01_ZpGdbd/ pJFRC49-10XUAS-IVS-eGFPKir2.1	ESC (SS01062) > Kir2.1	Ext Fig. 2
w^+; R48H08_p65ADZp/+; R29G11_ZpGdbd/ pJFRC49-10XUAS-IVS-eGFPKir2.1	L1, L2 (SS00797) > Kir2.1	Ext Fig. 2
w^+; R14A01_p65ADZp/+; R79H12_ZpGdbd/ pJFRC49-10XUAS-IVS-eGFPKir2.1	DNp01 (SS00727) > Kir2.1	Ext Fig. 2
w^+; R17A04_p65ADZp/+; R68A06_ZpGdbd/ pJFRC49-10XUAS-IVS-eGFPKir2.1	DNp01 (SS27721) > Kir2.1	Ext Fig. 2
w^+; VT063736_p65ADZp/+; R24A03_ZpGdbd/ pJFRC49-10XUAS-IVS-eGFPKir2.1	DNp02 (SS01053) > Kir2.1	Ext Fig. 2
w^+; VT063736_p65ADZp/+; VT017647_ZpGdbd/ pJFRC49-10XUAS-IVS-eGFPKir2.1	DNp02 (SS01554) > Kir2.1	Ext Fig. 2
w^+; R91C05_p65ADZp/+; R31B08_ZpGdbd/ pJFRC49-10XUAS-IVS-eGFPKir2.1	DNp03 (SS01081) > Kir2.1	Ext Fig. 2
w^+; R29F12_p65ADZp/+; R37G07_ZpGdbd/ pJFRC49-10XUAS-IVS-eGFPKir2.1	DNp03 (SS01596) > Kir2.1	Ext Fig. 2
w^+; VT032898_p65ADZp/+; VT048835_ZpGdbd/ pJFRC49-10XUAS-IVS-eGFPKir2.1	DNp04 (SS00934) > Kir2.1	Ext Fig. 2
w^+; R84B12_p65ADZp/+; VT048835_ZpGdbd/ pJFRC49-10XUAS-IVS-eGFPKir2.1	DNp04 (SS01080) > Kir2.1	Ext Fig. 2
w^+; VT047755_p65ADZp/+; VT003280_ZpGdbd/ pJFRC49-10XUAS-IVS-eGFPKir2.1	DNp05 (SS02612) > Kir2.1	Ext Fig. 2
w^+; VT019060_p65ADZp/+; VT003280_ZpGdbd/ pJFRC49-10XUAS-IVS-eGFPKir2.1	DNp05 (SS00865) > Kir2.1	Ext Fig. 2
w^+; VT019018_p65ADZp/+; VT017411_ZpGdbd/ pJFRC49-10XUAS-IVS-eGFPKir2.1	DNp06 (SS02256) > Kir2.1	Ext Fig. 2
w^+; BJD110D01_p65ADZp/+; R18G08_ZpGdbd/ pJFRC49-10XUAS-IVS-eGFPKir2.1	DNp11 (SS49010) > Kir2.1	Ext Fig. 2
w^+; BJD119F04_p65ADZp/+; R18G08_ZpGdbd/ pJFRC49-10XUAS-IVS-eGFPKir2.1	DNp11 (SS49024) > Kir2.1	Ext Fig. 2
w^+; R18G08_p65ADZp/+; BJD100H09_ZpGdbd/ pJFRC49-10XUAS-IVS-eGFPKir2.1	DNp11 (SS49051) > Kir2.1	Ext Fig. 2
w^+; VT048835_p65ADZp/+; VT017647_ZpGdbd/ pJFRC49-10XUAS-IVS-eGFPKir2.1	DNp02, DNp04 (SS01544) > Kir2.1	Ext Fig. 2

w+; VT017411_p65ADZp/+; VT017647_ZpGdbd/ pJFRC49-10XUAS-IVS-eGFPKir2.1	DNp02, DNp04, DNp06 (SS02292) > Kir2.1	Ext Fig. 2
w+; VT063736_p65ADZp/+; R24A03_ZpGdbd/ pJFRC28-10XUAS-IVS-GFP-p10	DNp02 (SS01053) > GFP	Fig. 3a-f, Ext Fig. 4c, 4i-j
w+; R18G08_p65ADZp/+; BJD100H09_ZpGdbd/ pJFRC28-10XUAS-IVS-GFP-p10	DNp11 (SS49051) > GFP	Fig. 3a-f, Ext Fig. 4c, 4i-j
w+; R17A04_p65ADZp/+; R68A06_ZpGdbd/ pJFRC28-10XUAS-IVS-GFP-p10	GF (SS27721) > GFP	Ext Fig. 4b-j
w+; VT032898_p65ADZp/+; VT048835_ZpGdbd/ pJFRC28-10XUAS-IVS-GFP-p10	DNp04 (SS00934) > GFP	Ext Fig. 4b-j
w*, hs-FLPG5.PEST; R47H03- p65ADZp/+; R72E01-ZpGdbd /10xUAS(FRT.stop)myr::smGdP-V5-THS 10xUAS(FRT.stop)myr::smGdP-FLAG}, 10xUAS(FRT.stop)myr::smGdP-HA	LC4>MCFO	Fig. 5a, Ext Fig. 8a, S. Video 3
hs-FLPG5.PEST; VT027704-p65ADZp/+; VT040569-ZpGdbd /10xUAS(FRT.stop)myr::smGdP-V5-THS 10xUAS(FRT.stop)myr::smGdP-FLAG}, 10xUAS(FRT.stop)myr::smGdP-HA	LC9>MCFO	Ext Fig. 8b, S. Video 4
hs-FLPG5.PEST; R64G10-p65ADZp/+; R35B06-ZpGdbd /10xUAS(FRT.stop)myr::smGdP-V5-THS 10xUAS(FRT.stop)myr::smGdP-FLAG}, 10xUAS(FRT.stop)myr::smGdP-HA	LC22>MCFO	Ext Fig. 8c, Ext Fig. 8f
hs-FLPG5.PEST; R19G02-p65ADZp /+; R75G12-ZpGdbd /10xUAS(FRT.stop)myr::smGdP-V5-THS 10xUAS(FRT.stop)myr::smGdP-FLAG}, 10xUAS(FRT.stop)myr::smGdP-HA	LPLC2>MCFO	Fig. 5b, Ext Fig. 8f
hs-FLPG5.PEST; R64G09- p65ADZp/+; R37H04-ZpGdbd /10xUAS(FRT.stop)myr::smGdP-V5-THS 10xUAS(FRT.stop)myr::smGdP-FLAG}, 10xUAS(FRT.stop)myr::smGdP-HA	LPLC1>MCFO	Ext. Fig. 8d
w*, UAS-myr::smGdP-HA, LexAop-myr::smGdP-V5; R49C04-LexA/VT063736-p65ADZp; GMR24A03-ZpGdbd /+	LC4>DNp02	Fig. 6c, Ext Fig.10a-b
w*, UAS-myr::smGdP-HA, LexAop-myr::smGdP-V5; R49C04-LexA/VT025392-p65ADZp; VT057247-ZpGdbd /+	LC4>DNp11	Fig. 6c, Ext Fig.10a-b
w*, UAS-myr::smGdP-HA, LexAop-myr::smGdP-V5; R49C04-LexA/VT032898-p65ADZp; VT048835-ZpGdbd /+	LC4>DNp04	Fig. 6c, Ext Fig.10a-b
w*, hs-FLPG5.PEST/UAS-myr::smGdP-HA; R49C04-LexA/VT063736-p65ADZp; LexAop-(FRT.stop)myr::GFP-2A-R, Brp-(RSRT.stop)myr::smGdP-V5/R24A03-ZpGdbd	LC4 sparse STaR > DNp02	Fig. 6e, 6g, Ext Fig. 10e, S. Video 5,6
w*, hs-FLPG5.PEST/UAS-myr::smGdP-HA; R49C04-LexA/VT025392-p65ADZp; LexAop-(FRT.stop)myr::GFP-2A-R, Brp-(RSRT.stop)myr::smGdP-V5/VT057247-ZpGdbd	LC4 sparse STaR > DNp11	Fig. 6f, 6h
w*, hs-FLPG5.PEST/UAS-myr::smGdP-HA; R75G12-LexA/R17A05-p65ADZp; LexAop-(FRT.stop)myr::GFP-2A-R, Brp-(RSRT.stop)myr::smGdP-V5/GMR68A06-ZpGdbd	LPLC2 sparse STaR > Giant Fiber	Fig. 6k, Ext Fig. 10f
w*; R68A06-LexA, LexAop-GFP/R19G02-p65ADZp; R75G12- ZpGdbd /UAS-P2X2, UAS-GFP	LPLC2>P2X2, GF-GFP	Fig 6m, Ext Fig. 12a-c
w*; R68A06-LexA, LexAop-GFP/R47H03-p65ADZp; R72E01- ZpGdbd /UAS-P2X2, UAS-GFP	LC4>P2X2, GF-GFP	Fig 6m, Ext Fig. 12a-c
w*; R68A06-LexA, LexAop-GFP/R22H02-p65ADZp; R20G06- ZpGdbd /UAS-P2X2, UAS-GFP	LC11>P2X2, GF-GFP	Ext Fig. 12a-c
