# Supplementary material for: Synaptic gradients transform object location to action
Source: Nature. 2023 Jan 4;613(7944):534–42. doi: 10.1038/s41586-022-05562-8 (PMC9849133; doi:10.1038/s41586-022-05562-8)
Supplement: Supplementary file 2 — Reporting Summary [file 41586_2022_5562_MOESM2_ESM.pdf]

nature portfolio

Corresponding author(s): Gwyneth Card, Larry Zipursky

Last updated by author(s): Oct 4, 2022

Reporting Summary

Nature Portfolio wishes to improve the reproducibility of the work that we publish. This form provides structure for consistency and transparency in reporting. For further information on Nature Portfolio policies, see our Editorial Policies and the Editorial Policy Checklist.

Statistics

For all statistical analyses, confirm that the following items are present in the figure legend, table legend, main text, or Methods section.

n/a	Confirmed	
☐	☒	The exact sample size (*n*) for each experimental group/condition, given as a discrete number and unit of measurement
☐	☒	A statement on whether measurements were taken from distinct samples or whether the same sample was measured repeatedly
☐	☒	The statistical test(s) used AND whether they are one- or two-sided<br>*Only common tests should be described solely by name; describe more complex techniques in the Methods section.*
☐	☒	A description of all covariates tested
☐	☒	A description of any assumptions or corrections, such as tests of normality and adjustment for multiple comparisons
☐	☒	A full description of the statistical parameters including central tendency (e.g. means) or other basic estimates (e.g. regression coefficient) AND variation (e.g. standard deviation) or associated estimates of uncertainty (e.g. confidence intervals)
☐	☒	For null hypothesis testing, the test statistic (e.g. *F*, *t*, *r*) with confidence intervals, effect sizes, degrees of freedom and *P* value noted<br>*Give P values as exact values whenever suitable.*
☒	☐	For Bayesian analysis, information on the choice of priors and Markov chain Monte Carlo settings
☒	☐	For hierarchical and complex designs, identification of the appropriate level for tests and full reporting of outcomes
☐	☒	Estimates of effect sizes (e.g. Cohen's *d*, Pearson's *r*), indicating how they were calculated

Our web collection on statistics for biologists contains articles on many of the points above.

Software and code

Policy information about availability of computer code

Data collection	FlyPEZ system (Williamson et al., 2018) was used to obtain high-speed videos of fly escape in order to quantitatively characterize differences in escape behavior. For visual stimulation, we used DMD projectors running at a refresh rate of 360 Hz, controlled by MATLAB using the Psychophysics Toolbox (See Methods, including both Behavioral assays and electrophysiological recordings). Immunofluorescence images were acquired using a Zeiss LSM 880 confocal microscope with Zen digital imaging software using oil-immersion 63x objective.
Data analysis	For the analysis of postural shifts and takeoff angles upon either optogenetic activation or looming stimulus presentation, we used a machine learning software, Animal Part Tracker (APT, a software package developed by the Branson Lab at Janelia) v0.3.4 (See Methods; associated code is available at https://zenodo.org/record/6366082). Whole-cell recording data were analyzed in MATLAB using custom written code or using Clampfit 11 software (Molecular Devices), and graphical representation was performed by using Prism 9.2.0 software (GraphPad). For anatomical analysis, confocal image stacks were exported to Imaris 9.7 for level adjustment, cropping and removal of signal in off-target brain regions and background noise, as well as 3D volume-reconstructions (see Methods for details of LC-DN colocalization and single-cell STaR data analysis). We annotated the FAFB serial section transmission electron microscopy volume using the CATMAID software (see Methods for details; all reconstructed neurons from the FAFB data set will be made available at https://fafb.catmaid.virtualflybrain.org/). To model the real-world receptive fields of the LC4 population we followed a previously established method (Morimoto et al. 2020, see Methods for details; the code is available at https://github.com/artxz/LC4_code). For "hemibrain" em dataset analysis, volumetric data of neurons and neuropils, as well as connectivity data and synapse locations were obtained from the neuPrint (hemibrain v1.1) database, (https://neuprint.janelia.org/) and have been processed with the natverse package for R (v4.0.3) using custom scripts (available at https://github.com/avaccari/DrosophilaVPNWiring). Detailed description of our analysis is available in the corresponding Methods section.

For manuscripts utilizing custom algorithms or software that are central to the research but not yet described in published literature, software must be made available to editors and reviewers. We strongly encourage code deposition in a community repository (e.g. GitHub). See the Nature Portfolio guidelines for submitting code & software for further information.

Data

Policy information about availability of data

All manuscripts must include a data availability statement. This statement should provide the following information, where applicable:

- Accession codes, unique identifiers, or web links for publicly available datasets
- A description of any restrictions on data availability
- For clinical datasets or third party data, please ensure that the statement adheres to our policy

All datasets generated and/or analyzed during the current study are available from the corresponding author on reasonable request

Human research participants

Policy information about studies involving human research participants and Sex and Gender in Research.

Reporting on sex and gender	*Use the terms sex (biological attribute) and gender (shaped by social and cultural circumstances) carefully in order to avoid confusing both terms. Indicate if findings apply to only one sex or gender; describe whether sex and gender were considered in study design whether sex and/or gender was determined based on self-reporting or assigned and methods used. Provide in the source data disaggregated sex and gender data where this information has been collected, and consent has been obtained for sharing of individual-level data; provide overall numbers in this Reporting Summary. Please state if this information has not been collected. Report sex- and gender-based analyses where performed, justify reasons for lack of sex- and gender-based analysis.*
Population characteristics	*Describe the covariate-relevant population characteristics of the human research participants (e.g. age, genotypic information, past and current diagnosis and treatment categories). If you filled out the behavioural & social sciences study design questions and have nothing to add here, write "See above."*
Recruitment	*Describe how participants were recruited. Outline any potential self-selection bias or other biases that may be present and how these are likely to impact results.*
Ethics oversight	*Identify the organization(s) that approved the study protocol.*

Note that full information on the approval of the study protocol must also be provided in the manuscript.

Field-specific reporting

Please select the one below that is the best fit for your research. If you are not sure, read the appropriate sections before making your selection.

☒ Life sciences ☐ Behavioural & social sciences ☐ Ecological, evolutionary & environmental sciences

For a reference copy of the document with all sections, see nature.com/documents/nr-reporting-summary-flat.pdf

Life sciences study design

All studies must disclose on these points even when the disclosure is negative.

Sample size	All sample sizes were chosen based on conventional standards used in our field. This value was determined on the basis of the expected magnitude of animal-to-animal variability, given published results and our own data.
Data exclusions	No data were excluded from the analysis except as noted for the behavior experiments (see Methods, "Behavioral Data Analysis").
Replication	For electrophysiological experiments (Fig. 3, Fig. 6m and corresponding Extended Data Figures) repeated measurements were taken from a given number of animals (both N and n values are indicated in the corresponding Figure Legends of panels).<br>For all other experiments, results were replicated in different individual flies across each dataset. We did not omit any replicates on the basis of the experimental results
Randomization	Animals were never arbitrarily assigned to treatment groups, and therefore there were no experiments where randomization could have been performed.
Blinding	The experimenter was not blind to genotype in this study. For electrophysiological recordings, the experimenter was guided by the cell body of a DN expressing GFP. For neuroanatomical experiments, the experimenter was able to see and recognize the morphology of individual neurons based on the fuorescence pattern.

Reporting for specific materials, systems and methods

We require information from authors about some types of materials, experimental systems and methods used in many studies. Here, indicate whether each material, system or method listed is relevant to your study. If you are not sure if a list item applies to your research, read the appropriate section before selecting a response.

Materials & experimental systems

n/a	Involved in the study
☐	☒ Antibodies
☒	☐ Eukaryotic cell lines
☒	☐ Palaeontology and archaeology
☐	☒ Animals and other organisms
☒	☐ Clinical data
☒	☐ Dual use research of concern

Methods

n/a	Involved in the study
☒	☐ ChIP-seq
☒	☐ Flow cytometry
☒	☐ MRI-based neuroimaging

Antibodies

Antibodies used

chicken anti-GFP (Abcam), Cat# ab13970
Rabbit anti-RFP (Clontech), Cat#632496
mouse anti-Bruchpilot (Developmental Studies Hybridoma Bank), Cat# nc82
Chicken anti-V5 (Bethyl Laboratories), Cat# A190-118A
Mouse anti-V5 (Abcam), cat# 27671
Rabbit anti-HA (Cell Signaling Technology), cat# 3724
Rat anti-HA (Roche), cat# 11867423001
Alexa Fluor 488 goat anti-chicken (Jackson ImmunoResearch Labs), Cat# 103-545-155
Alexa Fluor 488 Goat anti-Mouse IgG (Thermo Fisher), Cat# A28175
Alexa Fluor 568 Goat anti-Rabbit IgG (Thermo Fisher), Cat# A-11011
Alexa Fluor 568 Goat anti-Mouse IgG (Thermo Fisher), Cat# A-11004
Alexa Fluor® 647-AffiniPure Fab Fragment Goat Anti-Mouse IgG (Jackson ImmunoResearch Labs), Cat# 115-607-003
Alexa Fluor Plus 647 Goat anti-Rabbit IgG (Thermo Fisher), Cat# A32733TR
Alexa Fluor 647 Goat anti-Rat IgG (Thermo Fisher), Cat# A-21247

Validation

The anti-GFP antibody (Adcam), as well as anti-RFP (Clontech) are the standard antibodies used in the field for labeling Green Fluorescent Protein (GFP) and Red Fluorescent Protein (RFP). The secondary antibodies labeling GFP- and RFP- expressing cells (Alexa Fluor 488 goat anti-chicken and Alexa Fluor 568 goat anti-rabbit) were verified by us to target only those cells which express live GFP/ RFP fluorescence. The anti-bruchpilot (brp) antibody (nc82, DSHB) is a standard in the field as a background stain that labels presynaptic active zones. The secondary antibodies used for neuropil staining (Alexa Fluor 488 and 647 goat anti-mouse) was verified by us to reproduce the known patterns of neuropil borders (nc82 immunoreactivity) in published atlases (VirtualFlyBrain.org). Antibodies used for MCFO immunostaining (Fig. 5) (Rabbit anti-HA, Chicken anti-V5, as well as corresponding secondary antibodies) are validated for Drosophila melanogaster in Nern et al., 2015. Mouse anti-V5 antibody (Abcam) and secondary goat anti-mouse 488 IgG used to visualize presynaptic active zones labeled with Brp-V5 was validated in the original study describing the STaR methods (Chen et al., 2014). The same antibody was used to label membranes of LC4 in Fig. 6c. Rat anti-HA antibody (Roche) (as well as secondary antibody Alexa Fluor 647 goat anti-Rat IgG) used DNs (Fig. 6) were verified by us to target the cells of interest.

Animals and other research organisms

Policy information about studies involving animals; ARRIVE guidelines recommended for reporting animal research, and Sex and Gender in Research

Laboratory animals

Supplementary Table 2 provides detailed descriptions of fly genotypes used in each experiment and origins of transgenic stocks (including references to specific figure panels). Details on generation of transgenic stocks can be found in the corresponding Methods section

Wild animals

No wild animals were used in this study

Reporting on sex

Flies of both sexes were considered in all experiments unless specified otherwise; none of the findings of this study apply to only one sex and/or may be affected by sex. Both EM connectome datasets used in this study are obtained from female flies

Field-collected samples

No field samples were collected for this study

Ethics oversight

No ethical approval was required because experiments were performed on Drosophila melanogaster

Note that full information on the approval of the study protocol must also be provided in the manuscript.
